# Prevalence and Screening Tools of Intimate Partner Violence Among Pregnant and Postpartum Women: A Systematic Review and Meta-Analysis

**DOI:** 10.3390/ejihpe15080161

**Published:** 2025-08-15

**Authors:** Laura Brunelli, Flavia Pennisi, Antonio Pinto, Loredana Cella, Maria Parpinel, Silvio Brusaferro, Carlo Signorelli, Vincenzo Baldo, Vincenza Gianfredi

**Affiliations:** 1 Department of Medicine, University of Udine, 33100 Udine, Italy; laura.brunelli@uniud.it (L.B.); cella.loredana@spes.uniud.it (L.C.); maria.parpinel@uniud.it (M.P.); silvio.brusaferro@uniud.it (S.B.); 2 Accreditation, Quality and Clinical Risk Unit, Friuli Centrale Healthcare University Trust, 33100 Udine, Italy; 3 Faculty of Medicine, University Vita-Salute San Raffaele, 20132 Milan, Italy; pennisi.flavia@hsr.it (F.P.); signorelli.carlo@hsr.it (C.S.); 4 National PhD Programme in One Health Approaches to Infectious Diseases and Life Science Research, Department of Public Health, Experimental and Forensic Medicine, University of Pavia, 27100 Pavia, Italy; 5 Department of Cardiac, Thoracic, Vascular Sciences, and Public Health, University of Padua, 35122 Padua, Italy; vincenzo.baldo@unipd.it; 6 Department of Biomedical Sciences for Health, University of Milan, 20122 Milan, Italy; vincenza.gianfredi@unimi.it

**Keywords:** domestic violence, intimate partner violence, violence against women, pregnancy, postpartum, screening tools, public health

## Abstract

(1) Background: Domestic violence (DV), including intimate partner violence (IPV) during pregnancy and the puerperium, represents a major public health issue, significantly affecting maternal and child health. (2) Methods: This systematic review and meta-analysis, conducted according to PRISMA 2020 guidelines, aimed to identify screening tools used to detect DV and IPV among pregnant and postpartum women and to estimate DV prevalence. The protocol was published in PROSPERO in advance (CRD42023473392). (3) Results: A comprehensive literature search across PubMed, EMBASE, Scopus, and Web of Science was conducted on 1 January 2024, resulting in 34,720 records; 98 studies met the inclusion criteria. The included studies were conducted in over 40 countries, and most were cross-sectional. Commonly used screening tools included the WHO Women’s Health and Life Experiences Questionnaire, the Abuse Assessment Screen, and the WHO Violence Against Women Instrument. Meta-analyses showed that 10% of women experienced physical violence, 26% psychological violence, 9% sexual violence, 16% verbal violence, and 13% economic violence. The overall prevalence of IPV during pregnancy and the puerperium was 26%. Despite the widespread use of validated instruments, substantial heterogeneity was observed, underscoring the need for standardization. (4) Conclusion: These findings underline the urgent need to integrate routine IPV screening into maternal care pathways using validated, culturally adapted tools, ensuring women’s safety and confidentiality.

## 1. Introduction

Violence against women, as defined by the United Nations, encompasses “any act of gender-based violence that results in, or is likely to result in, physical, sexual, or mental harm or suffering to women, including threats of such acts, coercion or arbitrary deprivation of liberty, whether occurring in public or in private life” (United Nations, 1994). It represents one of the most common human rights violations worldwide, cutting across geographical, cultural, and socioeconomic boundaries (UN Women, 2024). When violence is perpetrated by a current or former intimate partner, it is referred to as intimate partner violence (IPV); more broadly, domestic violence (DV) includes abuse by any household member, such as parents or in-laws. This conceptual distinction is relevant for the selection of screening tools, the interpretation of prevalence estimates, and the design of appropriate interventions. Violence can take the form of physical, sexual, psychological, and economic violence (UN Women, 2024). When the violence is directed against pregnant or postpartum women, it can have short- and long-term physical, economic, and psychological consequences for both mother and child, preventing their full and equal participation in society. The significance of this issue is further magnified when considering the first 1000 days of life—a critical window for the child’s growth, development, and long-term health outcomes (Berg, 2016). Exposure to violence during this period can generate severe repercussions not only for individuals and families but also for societal structures, with lasting economic and social costs. Recognizing the global magnitude of this phenomenon, the international community has committed to eliminating all forms of violence against women and girls as a specific target within the Sustainable Development Goals (SDG No. 5—Gender Equality, Target 5.2) to be achieved by 2030 (United Nations, 2015).

The true extent of the phenomenon of violence against women, particularly IPV and DV, is difficult to measure due to significant underreporting, often driven by stigma, fear of retaliation, or sociocultural norms that discourage disclosure. Additionally, the absence of standardized international surveillance systems impedes a comprehensive understanding of the prevalence and nature of the phenomenon. Moreover, the conditions created by humanitarian (Meinhart et al., 2021), health, and environmental crises, such as the recent COVID-19 pandemic (Uzoho et al., 2023), conflict (Wirtz et al., 2014), and climate change (Boddy et al., 2024), appear to have further exacerbated gender-based violence by worsening pre-existing vulnerabilities and creating new and emerging threats. Considering the difficulty of self-reporting by victims of violence, particularly among pregnant and postpartum women, the systematic introduction of screening tools is essential to identify and provide timely support to pregnant and postpartum women at risk. These tools must be both effective and feasible for application within healthcare and community settings, where women can be reached during routine care encounters.

This systematic review aims to identify, describe, and appraise the screening tools available to detect intimate partner violence against women during pregnancy and the puerperium. Specifically, the review seeks to (I) summarize the characteristics and contexts of application of these tools, (II) assess the healthcare or community settings involved in their administration, and (III) explore reported prevalence rates of violence detected through these instruments. By doing so, the review intends to inform clinical practice and policy, supporting the development of effective strategies to protect maternal and child health during this vulnerable life stage.

## 2. Materials and Methods

### 2.1. Study Design and Protocol Registration

This systematic review and meta-analysis were conducted in accordance with the Preferred Reporting Items for Systematic Reviews and Meta-Analyses (PRISMA) guidelines (Page et al., 2021). The protocol was prospectively registered with the International Prospective Register of Systematic Reviews (PROSPERO; ID: CRD42023473392), ensuring methodological transparency and rigor.

### 2.2. Information Sources, Search Strategy, and Study Selection

A comprehensive literature search was conducted simultaneously across all selected databases—PubMed, EMBASE, Scopus, and Web of Science—on the 1st January 2024, ensuring the retrieval of all relevant studies available up to that date. The search strategy combined MeSH terms and keywords related to domestic violence and the target population. The full search strategy for each database is available in Supplementary Table S1.

The search was limited to studies published in English or Italian. Additional references were identified through manual screening of bibliographies of relevant articles.

Studies were eligible if they included pregnant women (across all trimesters) or women in the puerperium period (up to 40 days post-delivery) who were subjected to any form of domestic violence (DV), defined as physical, sexual, psychological abuse, or controlling behaviors, such as economic violence, perpetrated by an intimate partner, during pregnancy or the puerperium. Additionally, only studies using screening tools (e.g., questionnaires, checklists) designed to identify IPV during pregnancy or the puerperium were eligible.

The primary outcome was the prevalence of IPV among pregnant and puerperal women as assessed through the identified screening tools. Lastly, only original epidemiological research—descriptive, observational studies (cross-sectional, case–control, cohort) and interventional trials—published as peer-reviewed articles in English or Italian were included. Detailed inclusion/exclusion criteria are reported in Table 1.

### 2.3. Study Selection and Data Extraction

Study selection was performed in two steps. Firstly, two reviewers independently screened titles and abstracts of all retrieved records to identify studies meeting the eligibility criteria. Secondly, full-text articles were obtained and independently assessed for inclusion. Disagreements were resolved through discussion or consultation with a third reviewer.

Data extraction was conducted independently by two reviewers using a standardized, pre-piloted extraction form. Extracted data included authors, publication year, country, study period, study design, sample size, participants’ age and key characteristics, attrition rate, setting, type of screening tool used and validation, outcome measures and reported prevalence, adjustment variables, funding sources, and declared conflicts of interest. Any discrepancies were resolved through consensus or third-party adjudication. The authors of the original studies were contacted when data were missing or unclear. 

The classification of IPV subtypes, including distinctions between verbal and psychological abuse, was reported according to the terminology and categorization used in the original studies.

### 2.4. Quality Assessment

The methodological quality and risk of bias of included studies were independently assessed by two reviewers using the Newcastle–Ottawa Scale (NOS) adapted to each study design (Ottawa Hospital Research Institute, 2000). Any disagreements were resolved through discussion or with the involvement of a third reviewer.

### 2.5. Data Synthesis

Given the anticipated heterogeneity in study designs, populations, and screening tools, a narrative synthesis approach was adopted. Extracted data were synthesized to describe (I) the prevalence of IPV during pregnancy and the puerperium; (II) the characteristics of the screening tools utilized.

### 2.6. Statistical Analysis

A meta-analysis was conducted to estimate the event rate of violence during pregnancy. Analyses were performed within subgroups defined by the type of violence (physical, sexual, any, verbal, economic). For each study included in the meta-analysis, we systematically extracted the number of women who reported experiencing IPV (event count) and the total number of women analyzed (sample size). The selected effect size was the event rate (ER), defined as the proportion of women exposed to IPV within the study population. This measure was consistently used across all studies and all forms of IPV (physical, psychological, sexual, and any IPV). A meta-analysis was conducted for each category only when at least five studies were available. Some studies were included more than once when they provided distinct data points. For instance, studies reporting results separately for different time periods (e.g., before and during an intervention), different populations (e.g., by country), or different measurement instruments applied to the same sample were entered multiple times accordingly. Both fixed-effects and random-effects models were applied, depending on the degree of heterogeneity. Heterogeneity was assessed using the I^2^ statistic and interpreted as follows: not important (I^2^ < 25%), low (25% ≤ I^2^ < 50%), moderate (50% ≤ I^2^ < 75%), or high (I^2^ ≥ 75%) (Higgins et al., 2003). Publication bias was evaluated through visual inspection of funnel plot asymmetry and formally tested using Egger’s test, with a *p*-value < 0.10 considered indicative of potential bias (Egger et al., 1997). When bias was detected, the trim-and-fill method was used to adjust for the possible impact of missing studies. All statistical analyses were conducted using ProMeta ^®^ 3 software (Internovi, Cesena, Italy).

### 2.7. Sensitivity Analyses

To investigate the heterogeneity in IPV prevalence across different contexts, we conducted sensitivity analyses stratified by country income level, as defined by the World Bank, i.e., high, upper-middle, lower-middle, and low income, and by region, as defined by the World Health Organization (WHO): African, Americas, Eastern Mediterranean, European, South-East Asia, and Western Pacific (World Health Organization, n.d.). One study (Rasch et al., 2018) was excluded from the geographical analysis, because it was conducted in two countries (Tanzania and Vietnam) belonging to two different areas. These stratifications were performed to examine whether variations in IPV prevalence could be explained by economic or geographic factors. Sensitivity analyses were conducted for each form of IPV, namely, physical, psychological, sexual, and any IPV, but were limited to subgroups that included at least five studies to ensure the stability and reliability of the pooled estimates.

## 3. Results

### 3.1. Literature Search

**Figure 1:**
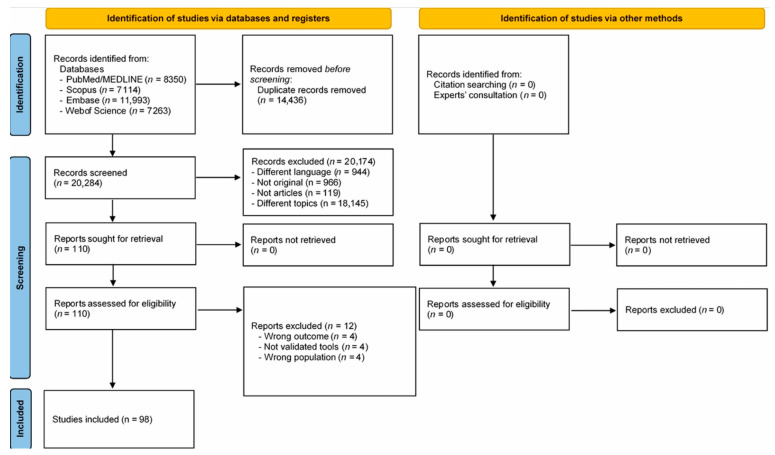
Flow diagram depicting the selection process.

A total of 34,720 records were identified by searching PubMed/MEDLINE (n = 8350), Scopus (n = 7114), EMBASE (n = 11,993), and Web of Science (n = 7263). No additional articles were included based on reference screening and expert consultation. After the preliminary exclusion of duplicates (n = 14,436), a total of 20,284 records were screened based on title and abstract. Based on the initial screening, records were excluded due to being non-original works (n = 966), conference papers (n = 119), written in different languages (n = 944), or focusing on unrelated topics or populations (n = 18,145), resulting in 110 records deemed eligible for inclusion. Based on full-text assessment, 12 records were excluded (not validated tool n = 4, wrong outcome n = 4, wrong population n = 4), resulting in 98 records included in the current systematic review (Table 2 and Table 3). The selection process is shown in Figure 1. Results are reported below according to the type of violence.

### 3.2. Results Categorized by Type of Violence

The majority of included studies assessed more than one IPV subtype; however, when disaggregated prevalence estimates were reported, we categorized them accordingly. Overlapping victimization is common in IPV, and this disaggregation does not imply mutually exclusive categories.

#### 3.2.1. Physical IPV

Out of the total 98 included articles, 80 studies assessing physical abuse during pregnancy were included in this systematic review. Study-level details, including year, country, study design, sample size, age, women’s status, setting, and used tools, are summarized in Table 2.

These studies were published between 2013 and 2023 and conducted in 39 countries across different regions, including Africa (Egypt, Ethiopia, Uganda, Nigeria, South Africa, Tanzania, Zimbabwe, Kenya, Namibia), Asia (Iran, Pakistan, India, Nepal, Bangladesh, Vietnam, China, Japan, Malaysia, Saudi Arabia, Jordan, Turkey, South Korea, Sri Lanka, Thailand, Timor-Leste), Europe (United Kingdom, Iceland, Denmark, Estonia, Norway, Sweden, Portugal, Spain, Greece, Belgium), North America (United States), South America (Brazil), and Oceania (Australia, Vanuatu). Regarding study design, the majority of studies were cross-sectional (CSS) (n = 64), followed by prospective cohort studies (PCSs) (n = 8), randomized controlled trial (RCT) (n = 2), case–control studies (CCSs) (n = 2), one mixed-methods study (MMS), and one quality improvement pilot study (QI). The sample sizes varied considerably, ranging from 65 to 7174 participants. The age of participants ranged from 13 to 50 years. Most studies focused on pregnant women (n = 68), whereas others focused on postpartum women (n = 5); in some cases, both pregnant and postpartum women were considered (n = 7). The data were collected in various healthcare and community settings, with studies conducted in hospital-based environments (n = 55) (including obstetric gynecologic departments, maternity wards, perinatal/antenatal, and postnatal clinics), primary healthcare centers (n = 17), and community-based settings (n = 7), and in one study a mobile app-based prenatal care system was implemented. Multiple validated tools were employed across studies to assess physical violence among pregnant and postpartum women. The most frequently used instrument was the WHO Women’s Health and Life Experiences Questionnaire (WHO-WHLEQ) (n = 23). This tool, developed and validated for cross-cultural applicability, was often adapted for specific national contexts, ensuring linguistic and cultural relevance. The Abuse Assessment Screen (AAS) was used in 17 studies (n = 17). The AAS was often adapted and validated for local contexts, for example, in Portuguese, Arabic, and Sinhalese versions, and applied both in hospital- and community-based settings. The WHO Violence Against Women Instrument (VAWI) was also adopted in studies (n = 13), indicating its broad international applicability for IPV assessment, including physical violence. Several studies (n = 8) also employed the Conflict Tactics Scale (CTS or CTS2), which evaluates conflict resolution strategies including physical aggression. This tool was used in countries like the USA, Iran, Bangladesh, and South Africa and is considered suitable for longitudinal studies and RCTs assessing intervention effects. Other validated instruments used less frequently include the Index of Spouse Abuse (ISA) (n = 4); the Woman Abuse Screening Tool (WAST) and its short form (n = 2); and the Composite Abuse Scale (CAS) (n = 3). The Hurt, Insult, Threaten, Scream (HITS) tool was used in three studies in Nigeria, India, and South Korea. The NorVold Abuse Questionnaire (NorAQ) was used in a European multinational study and in a study conducted in Sweden. Some studies also developed or used locally validated tools tailored to specific populations, such as the IPV During Pregnancy Questionnaire in Turkey and the Domestic Violence to Women Determination Scale (DVWDS). All tools were designed to detect physical abuse as a distinct category of intimate partner violence, frequently accompanied by measures of psychological and sexual violence. Overall, the wide adoption of culturally validated instruments across studies strengthens the reliability of reported prevalence estimates of physical violence during pregnancy and the puerperium. However, variations in tool structure, item formulation, recall periods, and thresholds for defining abuse may contribute to heterogeneity in findings.

In the meta-analysis, the fixed-effects model estimated an event rate (ES) of 0.15 (95% CI: 0.15–0.16, *p* < 0.001), based on a total of 63,429 participants. However, considerable heterogeneity was observed (I^2^ = 98.56%, *p* < 0.001). When the random-effects model was applied, the event rate decreased to 0.10 (95% CI: 0.08–0.12, *p* < 0.001). Publication bias was detected through visual inspection of the funnel plot and confirmed by Egger’s regression test (intercept = −6.83, *p* = 0.001). These findings are shown in Figure S1 (a: forest plot; b: funnel plot) and Table S2.

#### 3.2.2. Psychological IPV

A total of 66 studies reporting prevalence estimates of psychological or emotional abuse during pregnancy were incorporated into this systematic review. Study-level details, including year, country, study design, sample size, age, women’s status, setting, and used tools, are summarized in Table 3.

Published between 2013 and 2023, these studies were conducted in 36 countries across Africa (Egypt, Ethiopia, Nigeria, South Africa, Tanzania, Zimbabwe, Kenya, Namibia), Asia (Iran, Pakistan, India, Nepal, Bangladesh, Vietnam, China, Malaysia, Saudi Arabia, Jordan, Turkey, South Korea, Timor-Leste), Europe (United Kingdom, Iceland, Denmark, Estonia, Norway, Sweden, Portugal, Spain, Greece, Belgium, Malta), North America (United States), South America (Brazil), and Oceania (Australia, Vanuatu). Concerning methodological approaches, CSS predominated (n = 54), followed by PCS (n = 7), CCS (n = 2), RCT (n = 1), QI (n = 1), and one study employed an MMS. Participant sample sizes ranged widely, from 65 to 7174 individuals, with reported ages ranging from 13 to 50 years. The majority of investigations targeted pregnant women (n = 58); a smaller number focused exclusively on women in the postpartum period (n = 3), while others included both populations (n = 5). Data collection settings varied. Most studies were implemented in hospital-based environments (n = 43), including obstetrics and gynecology departments, maternity wards, and antenatal or postnatal clinics. Other settings included primary healthcare facilities (n = 16), community-based contexts (n = 6), and in one instance, a prenatal care model delivered via a mobile application. Several validated instruments were used across the included studies to assess psychological violence in pregnant and postpartum women, with the WHO-WHLEQ being the most frequently adopted (n = 22).

In the meta-analysis, the fixed-effects model estimated an ES of 0.30 (95% CI: 0.30–0.31, *p* < 0.001), based on a total of 55,361 participants. However, considerable heterogeneity was observed (I^2^ = 99.07%, *p* < 0.001). When the random-effects model was applied, the event rate decreased to 0.26 (95% CI: 0.22–0.31, *p* < 0.001). No publication bias was detected through visual inspection of the funnel plot, as confirmed by Egger’s regression test (intercept = −3.16, *p* = 0.256). These findings are shown in Figure S2 (a: forest plot; b: funnel plot) and Table S2.

**Table 2:** Characteristics of studies assessing physical IPV (n = 80), extracted from the total 98 included articles.

Author, Year	Country	Study Period	Study Design	Sample Size	Age in Years (Range or Mean and SD)	Woman Status	People Lost (Attrition Rate)	Setting	Tool Used to Assess the Outcome	Women Victims of Physical Violence (Prevalence)
(Abdollahi et al., 2015)	Iran	February–September 2010	PCS	1461	Mean 26.8 ± 5.8	Pre	n.a.	Primary healthcare center	WHO-WHLEQ	Physical 14.1%
(Abebe Abate et al., 2016)	Ethiopia	April 2014	CSS	282	Mean 27 ± 6.1; range 15–44	Pre	17	Community-based	WHO-WHLEQ	Physical 29.2%
(Abujilban et al., 2022)	Jordan	September–December 2014	CSS	247	Mean 27.3 ± 5.9	Pre and Pue	n.a.	Hospital-based	WHO-WHLEQ	Physical 31.2%
(Almeida et al., 2017)	Portugal	February–June 2012	CSS	852	Mean 30.69 ± 5.54; range 18–44	Pre	352	Hospital-based	WHO-WHLEQ	Physical 21.9%
(Antoniou & Iatrakis, 2019)	Greece	August–September 2009	CSS	546	Mean 32.95 ± 6.78	Pre	n.a.	Hospital-based	AAS	Physical injury (face 3.1%, abdomen 1.3%)
(Gómez Aristizábal et al., 2022)	Brazil	February 2010 and June 2011	PCS	1447	Mean 26.1 ± 5.4	Pre	317	Primary healthcare center	VAWI	Physical 12.5%
(Asiimwe et al., 2022)	Uganda	October 2018–February 2019	CSS	100	Mean 17.8 ± 1.26	Pre and Pue	n.a.	Hospital-based	VAWI	Physical 32.0%
(Atilla et al., 2023)	Turkey	September–October 2021	CSS	456	Mean 26.66 ± 5.45	Pre	24	Hospital-based	IPV During Pregnancy Questionnaire	Physical 6.6%
(Avcı et al., 2023)	Turkey	October 2017–August 2018	CSS	255	Mean 28.57 ± 6.17	Pre	n.a.	Primary healthcare center	DVWDS	Physical 14.6%
(Baǧcioǧlu et al., 2014)	Turkey	n.a.	CSS	317	Mean 27.4 ± 5.9	Pre	2	Hospital-based	AAS	Physical 5.3%
(Bahrami-Vazir et al., 2020)	Iran	2014	CSS	525	Mean 25.8 ± 5.1	Pre	25	Primary healthcare center	CTS2	Total IPV 67.0% of which: physical 22.0%
(Belay et al., 2019)	Ethiopia	February–August 2017	CSS	589	Mean 25; range 16–45	Pre	n.a.	Community-based	WHO-WHLEQ	Physical 9.2%
(Bernstein et al., 2016)	South Africa	March 2013–April 2014	CSS	623	Median age 28; range 18–44	Pre	n.a.	Primary healthcare center	VAWI	Physical 15.0%
(Bikinesi et al., 2017)	Namibia	n.a.	CSS	386	Mean 27.5 ± 6.8	Pre	n.a.	Primary healthcare center	WHO-WHLEQ	Physical 3.4%
(Boonnate et al., 2015)	Thailand	n.a.	CSS	230	Mean 28.98 ± 5.17	Pre	n.a.	Hospital-based	ISA	Physical 3.5%
(L. H. M. de Lima et al., 2016)	Brazil	May 2009–April 2010	CSS	359 (179 adolescents, 180 adults)	Adolescents: mean 17.5 ± 1.4; Adults: mean 26.8 ± 5.8	Pue	8	Hospital-based	AAS	Physical 3.3%
(Dinmohammadi et al., 2021)	Iran	August 2017	RCT	82 (41 intervention, 41 control)	Mean 27.55 ± 5.13 (intervention), 27.26 ± 4.46 (control)	Pre	8	Primary healthcare center	CTS2	Physical before 18.0% → after 7.0%
(Elkhateeb et al., 2021)	Egypt	n.a.	CSS	513	n.a.	Pre	37	Hospital-based	AAS	Physical 30.2%
(Farrokh-Eslamlou et al., 2014)	Iran	February–September 2012	CSS	313	Mean 27.9 ± 5.8; range 17–46	Pre	37	Hospital-based	AAS	Physical 10.2%
(Fekadu et al., 2018)	Ethiopia	March–May 2016	CSS	450	Mean 27 ± 4.5	Pre	n.a.	Hospital-based	WHO-WHLEQ	Physical 32.2%
(Ferdos et al., 2018)	Bangladesh	July 2015 to April 2016	CSS	443	<20 y 18.5%; 20–24 y 43.9%; 25–35 y 37.6%	Pue	43	Hospital-based	CTS	Physical 39.0%
(Field et al., 2018)	South Africa	November 2011–August 2012	MMS	376, 95 case notes analyzed qualitatively	Age categories: 18–24 years (39%), 25–29 years (30%), >29 years (31%)	Pre	186	Hospital-based	CTS2	Physical 76.0%
(Fonseca-Machado et al., 2015)	Brazil	May 2012–May 2013	CSS	358	Mean 25.0 ± 6.3; range 15–43	Pre	n.a.	Hospital-based	WHO-WHLEQ	Physical 36.5%
(Gebrekristos et al., 2023)	South Africa	July 2017–April 2018	CSS	90	Mean 17.5 ± 1.4; range 14–19	Pre and Pue	29	Hospital-based	CTS	Physical 16.7%
(Gharacheh et al., 2015)	Iran	July–December 2012	CSS	328	Abused: mean 26.25 ± 4.12); Non-abused: mean 27.14 ± 4.29)	Pue	13	Primary healthcare center	AAS	Physical 26.0%
(Gul et al., 2013)	Pakistan	April 2010–March 2011	CSS	129	Mean 31.42 ± 7.02; range 15–50	Pre	n.a.	Hospital-based	AAS	Physical 35.7%
(Ilori et al., 2023)	Nigeria	March–September 2019	CSS	240	Mean 30.7 ± 5.5	Pre	n.a.	Hospital-based	CAS	Physical 39.3%
(Islam et al., 2021)	Bangladesh	October 2015–January 2016	CSS	426	Mean 26.28 ± 5.87; range 15–49	Pue	27	Primary healthcare center	WHO-WHLEQ	Physical 35.2%
(Iyengar et al., 2021)	United Kingdom	3 months in 2016	CSS	120	Mean 25.22 ± 4.93	Pre	n.a.	Hospital-based	WHO-WHLEQ	Physical + sexual 57.0%
(Kana et al., 2020)	Nigeria	January 2017–April 2019	CSS	293	Mean 28.8 ± 5.9 in IPV-exposed group, 29.2 ± 5.7 in unexposed group	Pre	35	Hospital-based	CTS	Physical 34.1%
(Khaironisak et al., 2017)	Malaysia	March–August 2015	CSS	1200	Mean 29.07 ± 5.39	Pre	n.a.	Hospital-based	WHO-WHLEQ	Physical 12.9%
(Khatlani et al., 2023)	Pakistan	February–May 2014	CCS	795 women (256 cases with stillbirths, 539 controls with live births)	Mean 29.6 ± 5.9 in stillbirth group; mean 28.7 ± 5.7 in live birth group	Pre	n.a.	Community-based	WHO-WHLEQ	Physical 9.94%
(Kita et al., 2017)	Japan	July 2013–July 2014	PCS	453	Mean 32.1 ± 4.9; range 19–46	Pre	502	Hospital-based	WAST-Short	Physical 3.3%
(Kita et al., 2016)	Japan	July 2013–July 2014	PCS	562	Mean 32.2 ± 4.9; range 19–46	Pre	393	Hospital-based	ISA	Physical 2.5%
(Koirala, 2022)	Nepal	June–September 2020	CSS	220	Mean 30.18 ± 5.70	Pre	n.a.	Hospital-based	VAWI	Physical 28.6%
(Krishnamurti et al., 2021)	USA	January–May 2020	QI	959 (552 before shelter-in-place, 407 during shelter-in-place)	n.a.	Pre	n.a.	Mobile app	CDC BRFSS (for physical and sexual IPV), WEB (for psychological IPV)	Physical: before 0.4% (552), during: 0.5% (407)
(Lee et al., 2023)	South Korea	2020–2021	CSS	5616	Range 16–48	Pre and Pue	337	Primary healthcare center	HITS	physical 0.3%
(L. da S. Lima et al., 2020)	Brazil	September–October 2018	CSS	65	Mean 23.88; range 15–42	Pre	n.a.	Primary healthcare center	WHO-WHLEQ	Physical 18.5%
(Luhumyo et al., 2020)	Kenya	April–June 2017	CSS	369	Median age 25 (IQR: 21–31)	Pre	n.a.	Hospital-based	VAWI	Physical 22.8%
(Lukasse et al., 2014)	Belgium, Iceland, Denmark, Estonia, Norway, Sweden	March 2008–August 2010	PCS	7174	n.a.	Pre	n.a.	Hospital-based	NorAQ	Physical 2.2%
(Mahenge et al., 2013)	Tanzania	December 2011–April 2012	CSS	1180	Mean 29.0; range 17–43	Pre	20	Hospital-based	CTS	Physical 18.0%
(S. Martin-de-las-Heras et al., 2019)	Spain	February–June 2010	PCS	779	Mean 29.9 ± 5.6	Pre	214	Hospital-based	ISA	Physical 3.6%
(Stella Martin-de-las-Heras et al., 2015)	Spain	n.a.	CSS	779	Mean 29.9 ± 5.6	Pre	153	Hospital-based	ISA	Physical 3.6%
(McKelvie et al., 2021)	Vanuatu	May–July 2019	CSS	188	Mean 25.7 ± 5.4	Pre	4	Hospital-based	VAWI	Physical 10.6%
(Mohamed et al., 2013)	Saudi Arabia	October 2012–February 2013	CSS	404	Mean 31.19 ± 7.36	Pre	12	Primary healthcare center	WAST	Physical 28.0%
(Musa et al., 2020)	Ethiopia	November 2018–April 2019	CSS	648	n.a.	Pre	n.a.	Hospital-based	WHO-WHLEQ	Physical 25.93%
(Muzrif et al., 2018)	Sri Lanka	April–December 2014	CSS	2088	Mean 29.63 ± 5.57); 53.9% aged 16–30 years, 46.1% aged 31–44 years	Pre	87	Hospital-based	AAS	Physical 6.4%
(Naghizadeh et al., 2021)	Iran	May–August 2020	CSS	250	Mean 30.57 ± 5.87	Pre	n.a.	Hospital-based	WHO-WHLEQ	Physical 4.8%
(Nhi et al., 2019)	Vietnam	May 2014–August 2015	PCS	1274	Mean 26; range 16–46	Pre and Pue	63	Hospital-based	WHO-WHLEQ	Physical 3.5%
(Njoku et al., 2021)	Nigeria	January–March 2017	CSS	400	Mean 30.1 ± 2.47; range 20–45	Pre	n.a.	Hospital-based	AAS	Physical 44.8%
(Okunola et al., 2021)	Nigeria	March 2019 and September 2019	PCS	363	Mean 30 ± 5.3	Pre	0	Hospital-based	Ongoing abuse screen	Physical 3.6%
(Omoronyia et al., 2020)	Nigeria	n.a.	CSS	250	29.7 ± 6.1	Pre	n.a.	Hospital-based	CAS	Physical 26.8%
(Priya et al., 2019)	India	December 2013–February 2015	CSS	165	23.8 ± 3.8	Pre	n.a.	Community-based	HITS	Physical 60.0%
(Pun et al., 2019)	Nepal	June 2015–September 2016	PCS	1381	Age categories: 15–19 (5.6%), 20–24 (42.8%), 25–29 (37.6%), ≥30 (14.0%)	Pre	623	Hospital-based	VAWI	Physical 2.5%
(Pun et al., 2018)	Nepal	November 2014–November 2015	CSS	1011	Mean 24.4 ± 4.0	Pre	28	Hospital-based	AAS	Physical 4.0%; physical or psychological 6.1%
(Rasch et al., 2018)	Tanzania and Vietnam	n.a.	CSS	2425 (1116 in Tanzania, 1309 in Vietnam)	n.a.	Pre	n.a.	Hospital-based	VAWI	Tanzania: physical 6.0%; Vietnam: physical 3.5%
(S. Rees et al., 2017)	Timor-Leste	June 2013–September 2014	CSS	1672	Age groups: <20 years (8.4%), 20–24 (34.0%), 25–29 (34.4%), 30–34 (16.3%), ≥35 (6.8%)	Pre	2	Community-based	VAWI	Physical 6.2%
(S. J. Rees et al., 2016)	Timor-Leste	May 2014–January 2015	CSS	1672	Age groups: 20 years: 141 (8.4%); 20–24 (34.0%); 25–29 (34.4%); 30–34 (16.3%); ≥35 (6.8%)	Pre	2	Hospital-based	WHO-WHLEQ	Physical 6.2%
(S. V. O. Ribeiro et al., 2019)	Brazil	2010–2013	PCS	1139	n.a.	Pre	n.a.	Primary healthcare center	VAWI	Physical 12.1%
(M. R. C. Ribeiro et al., 2017)	Brazil	February 2010–June 2011	CSS	1446 (São Luís), 1378 (Ribeirão Preto)	n.a.	Pre	1	Primary healthcare center	VAWI	Physical 12.4%
(Samal & Poornesh, 2022)	India	October–November 2016	CSS	200	Range 19–40	Pre	n.a.	Hospital-based	AAS	Physical 0.5%
(Sánchez et al., 2023)	Brazil	July 2019–September 2021	CSS	600	Mean 27.0 ± 8.58; range 13–47	Pre and Pue	n.a.	Hospital-based	AAS, WAST, HITS	Physical 2.3%
(Sapkota et al., 2021)	Nepal	June–August 2018	RCT	140	Mean 25.3 ± 5.4	Pre	3	Hospital-based	AAS	Physical 12.1%
(Shamu et al., 2014)	Zimbabwe	May–September 2011	CSS	1951	n.a.	Pre and Pue	n.a.	Hospital-based	WHO-WHLEQ	Physical 5.8%
(Shamu et al., 2013)	Zimbabwe	May–September 2011	CSS	2042	Mean 26 ± 5.71; range 15–48	Pre	59	Primary healthcare center	WHO-WHLEQ	Physical 15.9%; physical and/or sexual 46.2%
(Shannon et al., 2016)	USA	August 2005–October 2007	CSS	77	Mean 24.96 ± 3.83	Pre	n.a.	Hospital-based	NVAWS, CTS2, PMWI	Physical 32.5%
(Shrestha et al., 2016)	Nepal	September–November 2015	CSS	404	Mean 25.5 ± 4.3; 43.8% <25	Pre	n.a.	Hospital-based	WHO-WHLEQ	Physical 3.2%
(Silva et al., 2022)	Brazil	August–October 2017	CSS	327	Not explicitly reported; categorized as ≤40 years and >40 years	Pre	n.a.	Hospital-based	VAWI	Physical 7.2%
Silva, 2019 (Silva & Leite, 2019)	Brazil	August–October 2017	CSS	330	Not explicitly reported; categorized as 14–19 years and ≥20 years	Pre	n.a.	Hospital-based	VAWI	Physical 7.6%
(Sobhani et al., 2018)	Iran	September–December 2014	CSS	402	Mean 28.24 ± 5.91; range 13–44	Pre	n.a.	Hospital-based	WHO-WHLEQ	Physical 10.2%
(Sulaiman et al., 2021)	Nigeria	November 2018–August 2019	CSS	403	Mean 33 ± 4.9	Pre	8	Hospital-based	HITS	Physical 22.1%
(Takelle et al., 2023)	Ethiopia	May–June 2022	CSS	473	Mean 28.18 ± 5.28; range 18–41	Pre	12	Hospital-based	WHO-WHLEQ	Physical 4.0%
(Utaile et al., 2023)	Ethiopia	July–October 2020	CSS	1535	Mean 26.3 ± 4.7	Pre	n.a.	Community-based	WHO-WHLEQ	Physical 34.0%
(Velasco et al., 2014)	Spain	2009	CSS	779	Mean 29.9 ± 5.6	Pre	n.a.	Hospital-based	AAS, ISA	AAS: physical 1.7%; ISA: physical 3.6%
(Wangel et al., 2016)	Sweden	March–November 2008	CSS	1003	Age groups: <25 years (11.2%), 25–29 (32%), 30–35 (43.2%), >35 (13.7%)	Pre	22	Hospital-based	NorAQ	Physical 14.2%
(Watson & Taft, 2013)	Australia	April 2002–March 2004	CCS	1726	n.a.	Pre	54	Hospital-based	CAS	Physical 9.9%
(Wokoma et al., 2014)	United Kingdom	January 2011–November 2012	CSS	507	TOP group (women requesting termination of pregnancy) mean 24.4, ANC group (antenatal care) mean 28.8	Pre	55	Hospital-based	AAS	Physical: TOP 4.7%, ANC 0.9%
(Zapata-Calvente et al., 2022)	Spain	January 2017–March 2019	CSS	592	Mean 31.82 ± 5.61	Pre	138	Primary healthcare center	WAST-Short, AAS	Physical 5.4%
(Zheng et al., 2020)	China	July–October 2019	CSS	813	Mean 28.98 ± 4.52	Pre	n.a.	Community-based	AAS	Physical 0.98%
(Zou et al., 2015)	China	October 2006–February 2007	CSS	223 (86 in DV group, 137 in non-DV group)	DV group: 27.8 ± 2.7; non-DV group: 27.2 ± 3.0	Pre	23	Primary healthcare center	AAS	Physical + psychological + sexual 2.3%

AAS: Abuse Assessment Screen; ANC: antenatal care; CAS: Composite Abuse Scale; CDC BRFSS: Centers for Disease Control—Behavioral Risk Factor Surveillance System; CCS: case–control Study; CSS: cross-sectional study; CTS: Conflict Tactics Scale; CTS2: Revised Conflict Tactics Scale; DV: domestic violence; DVWDS: Domestic Violence to Women Determination Scale; HITS: Hurt, Insult, Threaten, Scream; IPV: intimate partner violence; IQR: Interquartile Range; ISA: Index of Spouse Abuse; MMS: mixed-methods study; NorAQ: NorVold Abuse Questionnaire; NVAWS: National Violence Against Women Survey; PCS: prospective cohort study; PMWI: Psychological Maltreatment of Women Inventory; Pre: pregnancy; Pue: puerperium; QI: quality improvement pilot study; RCT: randomized controlled trial; SD: Standard Deviation; TOP: termination of pregnancy; USA: United States of America; VAWI: Violence Against Women Instrument; WAST: Woman Abuse Screening Tool; WAST-Short: Woman Abuse Screening Tool—Short Version; WEB: Women’s Experience with Battering; WHO-WHLEQ: World Health Organization—Women’s Health and Life Experiences Questionnaire.

**Table 3:** Characteristics of studies assessing psychological IPV (n = 66), extracted from the total 98 included articles.

Author, Year	Country	Study Period	Study Design	Sample Size	Age in Years (Range or Mean and SD)	Woman Status	People Lost (Attrition Rate)	Setting	Tool Used to Assess the Outcome	Women Victims of Psychological Violence (Prevalence)
(Abebe Abate et al., 2016)	Ethiopia	April 2014	CSS	282	Mean 27 ± 6.1; range 15–44	Pre	17	Community-based	WHO-WHLEQ	Psychological 16.3%
(Abujilban et al., 2022)	Jordan	September–December 2014	CSS	247	Mean 27.3 ± 5.9	Pre and Pue	n.a.	Hospital-based	WHO-WHLEQ	Psychological 66.0%
(Almeida et al., 2017)	Portugal	February–June 2012	CSS	852	Mean 30.69 ± 5.54; range 18–44	Pre	352	Hospital-based	WHO-WHLEQ	Psychological 43.2%
(Antoniou & Iatrakis, 2019)	Greece	August–September 2009	CSS	546	Mean 32.95 ± 6.78	Pre	n.a.	Hospital-based	AAS	Psychological 2.8%
(Gómez Aristizábal et al., 2022)	Brazil	February 2010 and June 2011	PCS	1447	Mean 26.1 ± 5.4	Pre	317	Primary healthcare center	VAWI	Psychological 48.5%
(Atilla et al., 2023)	Turkey	September–October 2021	CSS	456	Mean 26.66 ± 5.45	Pre	24	Hospital-based	IPV During Pregnancy Questionnaire	Psychological 33.3%
(Avcı et al., 2023)	Turkey	October 2017–August 2018	CSS	255	Mean 28.57 ± 6.17	Pre	n.a.	Primary healthcare center	DVWDS	Psychological 38.8%
(Bahrami-Vazir et al., 2020)	Iran	2014	CSS	525	Mean 25.8 ± 5.1	Pre	25	Primary healthcare center	CTS2	Total IPV 67.0% of which: psychological 58.0%
(Belay et al., 2019)	Ethiopia	February–August 2017	CSS	589	Mean 25; range 16–45	Pre	n.a.	Community-based	WHO-WHLEQ	Psychological 14.6%
(Bernstein et al., 2016)	South Africa	March 2013–April 2014	CSS	623	Median age 28; range 18–44	Pre	n.a.	Primary healthcare center	VAWI	Psychological 15.0%
(Bikinesi et al., 2017)	Namibia	n.a.	CSS	386	Mean 27.5 ± 6.8	Pre	n.a.	Primary healthcare center	WHO-WHLEQ	Psychological 7.0%
(Debono et al., 2017)	Malta	October 2014–January 2015	CSS	300	Mean 30.7; range: 18–43	Pre	80	Hospital-based	VAWI	Psychological 15.0%
(Dinmohammadi et al., 2021)	Iran	August 2017	RCT	82 (41 intervention, 41 control)	Mean 27.55 ± 5.13 (intervention), 27.26 ± 4.46 (control)	Pre	8	Primary healthcare center	CTS2	Psychological before 56.0% → after 36.0%
(Elkhateeb et al., 2021)	Egypt	n.a.	CSS	513	n.a.	Pre	37	Hospital-based	AAS	Psychological 45.4%
(Farrokh-Eslamlou et al., 2014)	Iran	February–September 2012	CSS	313	Mean 27.9 ± 5.8; range 17–46	Pre	37	Hospital-based	AAS	Psychological 43.5%
(Fekadu et al., 2018)	Ethiopia	March–May 2016	CSS	450	Mean 27 ± 4.5	Pre	n.a.	Hospital-based	WHO-WHLEQ	Psychological 57.8%
(Field et al., 2018)	South Africa	November 2011–August 2012	MMS	376, 95 case notes analyzed qualitatively	Age categories: 18–24 years (39%), 25–29 years (30%), >29 years (31%)	Pre	186	Hospital-based	CTS2	Psychological 81.0%
(Fonseca-Machado et al., 2015)	Brazil	May 2012–May 2013	CSS	358	Mean 25.0 ± 6.3; range 15–43	Pre	n.a.	Hospital-based	WHO-WHLEQ	Psychological 95.2%
(Gebrekristos et al., 2023)	South Africa	July 2017–April 2018	CSS	90	Mean 17.5 ± 1.4; range 14–19	Pre and Pue	29	Hospital-based	CTS	Psychological 36.7%
(Gharacheh et al., 2015)	Iran	July–December 2012	CSS	328	Abused: mean 26.25 ± 4.12); Non-abused: mean 27.14 ± 4.29)	Pue	13	Primary healthcare center	AAS	Psychological 88.4%
(Gul et al., 2013)	Pakistan	April 2010–March 2011	CSS	129	Mean 31.42 ± 7.02; range 15–50	Pre	n.a.	Hospital-based	AAS	Psychological 46.5%
(Ilori et al., 2023)	Nigeria	March–September 2019	CSS	240	Mean 30.7 ± 5.5	Pre	n.a.	Hospital-based	CAS	Psychological 40.2%
(Islam et al., 2021)	Bangladesh	October 2015–January 2016	CSS	426	Mean 26.28 ± 5.87; range 15–49	Pue	27	Primary healthcare center	WHO-WHLEQ	Psychological 65.0%
(Iyengar et al., 2021)	United Kingdom	3 months in 2016	CSS	120	Mean 25.22 ± 4.93	Pre	n.a.	Hospital-based	WHO-WHLEQ	Psychological 43.0%
(Kana et al., 2020)	Nigeria	January 2017–April 2019	CSS	293	Mean 28.8 ± 5.9 in IPV-exposed group, 29.2 ± 5.7 in unexposed group	Pre	35	Hospital-based	CTS	Psychological 51.2%
(Khaironisak et al., 2017)	Malaysia	March–August 2015	CSS	1200	Mean 29.07 ± 5.39	Pre	n.a.	Hospital-based	WHO-WHLEQ	Psychological 29.8%
(Khatlani et al., 2023)	Pakistan	February–May 2014	CCS	795 women (256 cases with stillbirths, 539 controls with live births)	Mean 29.6 ± 5.9 in stillbirth group; mean 28.7 ± 5.7 in live birth group	Pre	n.a.	Community-based	WHO-WHLEQ	Psychological 38.87%
(Koirala, 2022)	Nepal	June–September 2020	CSS	220	Mean 30.18 ± 5.70	Pre	n.a.	Hospital-based	VAWI	Psychological 30.9%
(Krishnamurti et al., 2021)	USA	January–May 2020	QI	959 (552 before shelter-in-place, 407 during shelter-in-place)	n.a.	Pre	n.a.	Mobile app	CDC BRFSS (for physical and sexual IPV), WEB (for psychological IPV)	Psychological before 1.0% (552), during 0.7% (407)
(Lee et al., 2023)	South Korea	2020–2021	CSS	5616	Range 16–48	Pre and Pue	337	Primary healthcare center	HITS	Psychological 3.4%
(L. da S. Lima et al., 2020)	Brazil	September–October 2018	CSS	65	Mean 23.88; range 15–42	Pre	n.a.	Primary healthcare center	WHO-WHLEQ	Psychological 40.0%
(Luhumyo et al., 2020)	Kenya	April–June 2017	CSS	369	Median age 25 (IQR: 21–31)	Pre	n.a.	Hospital-based	VAWI	Psychological 27.4%
(Lukasse et al., 2014)	Belgium, Iceland, Denmark, Estonia, Norway, Sweden	March 2008–August 2010	PCS	7174	n.a.	Pre	n.a.	Hospital-based	NorAQ	Psychological 2.7%
(S. Martin-de-las-Heras et al., 2019)	Spain	February–June 2010	PCS	779	Mean 29.9 ± 5.6	Pre	214	Hospital-based	ISA	Psychological 21.0%
(Stella Martin-de-las-Heras et al., 2015)	Spain	n.a.	CSS	779	Mean 29.9 ± 5.6	Pre	153	Hospital-based	ISA	Psychological 21.0%
(McKelvie et al., 2021)	Vanuatu	May–July 2019	CSS	188	Mean 25.7 ± 5.4	Pre	4	Hospital-based	VAWI	Psychological 39.1%
(Mohamed et al., 2013)	Saudi Arabia	October 2012–February 2013	CSS	404	Mean 31.19 ± 7.36	Pre	12	Primary healthcare center	WAST	Psychological 39%
(Musa et al., 2020)	Ethiopia	November 2018–April 2019	CSS	648	n.a.	Pre	n.a.	Hospital-based	WHO-WHLEQ	Psychological 25.62%
(Naghizadeh et al., 2021)	Iran	May–August 2020	CSS	250	Mean 30.57 ± 5.87	Pre	n.a.	Hospital-based	WHO-WHLEQ	Psychological 32.8%
(Nhi et al., 2019)	Vietnam	May 2014–August 2015	PCS	1274	Mean 26; range 16–46	Pre and Pue	63	Hospital-based	WHO-WHLEQ	Psychological 32.3%
(Njoku et al., 2021)	Nigeria	January–March 2017	CSS	400	Mean 30.1 ± 2.47; range 20–45	Pre	n.a.	Hospital-based	AAS	Psychological 34.3%
(Okunola et al., 2021)	Nigeria	March 2019 and September 2019	PCS	363	Mean 30 ± 5.3	Pre	0	Hospital-based	Ongoing abuse screen	Psychological 3.9%
(Omoronyia et al., 2020)	Nigeria	n.a.	CSS	250	29.7 ± 6.1	Pre	n.a.	Hospital-based	CAS	Psychological 51.2%; psychological + sexual 10.8%
(Pun et al., 2019)	Nepal	June 2015–September 2016	PCS	1381	Age categories: 15–19 (5.6%), 20–24 (42.8%), 25–29 (37.6%), ≥30 (14.0%)	Pre	623	Hospital-based	VAWI	Psychological 5.2%
(Pun et al., 2018)	Nepal	November 2014–November 2015	CSS	1011	Mean 24.4 ± 4.0	Pre	28	Hospital-based	AAS	Psychological or physical 6.1%
(Rasch et al., 2018)	Tanzania and Vietnam	n.a.	CSS	2425 (1116 in Tanzania, 1309 in Vietnam)	n.a.	Pre	n.a.	Hospital-based	VAWI	Tanzania: psychological 22.8%; Vietnam: psychological 32.2%
(S. Rees et al., 2017)	Timor-Leste	June 2013–September 2014	CSS	1672	Age groups: <20 years (8.4%), 20–24 (34.0%), 25–29 (34.4%), 30–34 (16.3%), ≥35 (6.8%)	Pre	2	Community-based	VAWI	Psychological 30.6%
(S. J. Rees et al., 2016)	Timor-Leste	May 2014–January 2015	CSS	1672	Age groups: 20 years 141 (8.4%); 20–24 (34.0%); 25–29 (34.4%); 30–34 (16.3%); ≥35 (6.8%)	Pre	2	Hospital-based	WHO-WHLEQ	Psychological 30.6%
(S. V. O. Ribeiro et al., 2019)	Brazil	2010–2013	PCS	1139	n.a.	Pre	n.a.	Primary healthcare center	VAWI	Psychological 47.3%
(M. R. C. Ribeiro et al., 2017)	Brazil	February 2010–June 2011	CSS	1446 (São Luís), 1378 (Ribeirão Preto)	n.a.	Pre	1	Primary healthcare center	VAWI	Psychological 48.4%
(Samal & Poornesh, 2022)	India	October–November 2016	CSS	200	Range 19–40	Pre	n.a.	Hospital-based	AAS	Psychological 1.0%
(Shamu et al., 2014)	Zimbabwe	May–September 2011	CSS	1951	n.a.	Pre and Pue	n.a.	Hospital-based	WHO-WHLEQ	Psychological 18.0%
(Shamu et al., 2013)	Zimbabwe	May–September 2011	CSS	2042	Mean 26 ± 5.71; range 15–48	Pre	59	Primary healthcare center	WHO-WHLEQ	Psychological 44.0%
(Shannon et al., 2016)	USA	August 2005–October 2007	CSS	77	Mean 24.96 ± 3.83	Pre	n.a.	Hospital-based	NVAWS, CTS2, PMWI	Psychological 71.4%
(Shrestha et al., 2016)	Nepal	September–November 2015	CSS	404	Mean 25.5 ± 4.3; 43.8% <25	Pre	n.a.	Hospital-based	WHO-WHLEQ	Psychological 16.6%
(Silva et al., 2022)	Brazil	August–October 2017	CSS	327	Not explicitly reported; categorized as ≤40 years and >40 years	Pre	n.a.	Hospital-based	VAWI	Psychological 16.8%
(Silva & Leite, 2019)	Brazil	August–October 2017	CSS	330	Not explicitly reported; categorized as 14–19 years and ≥20 years	Pre	n.a.	Hospital-based	VAWI	Psychological 16.1%
(Sobhani et al., 2018)	Iran	September–December 2014	CSS	402	Mean 28.24 ± 5.91; range 13–44	Pre	n.a.	Hospital-based	WHO-WHLEQ	Psychological 45.5%
(Takelle et al., 2023)	Ethiopia	May–June 2022	CSS	473	Mean 28.18 ± 5.28; range 18–41	Pre	12	Hospital-based	WHO-WHLEQ	Psychological 6.3%
(Utaile et al., 2023)	Ethiopia	July–October 2020	CSS	1535	Mean 26.3 ± 4.7	Pre	n.a.	Community-based	WHO-WHLEQ	Psychological 34.6%
(Velasco et al., 2014)	Spain	2009	CSS	779	Mean 29.9 ± 5.6	Pre	n.a.	Hospital-based	AAS, ISA	AAS: psychological 4.8%; ISA: psychological 21.0%
(Watson & Taft, 2013)	Australia	April 2002–March 2004	CCS	1726	n.a.	Pre	54	Hospital-based	CAS	Psychological 12.8%
(Wokoma et al., 2014)	United Kingdom	January 2011–November 2012	CSS	507	TOP group (women requesting termination of pregnancy) mean 24.4, ANC group (antenatal care) mean 28.8	Pre	55	Hospital-based	AAS	Psychological: TOP 9.9%, ANC 1.8%
(Zapata-Calvente et al., 2022)	Spain	January 2017–March 2019	CSS	592	Mean 31.82 ± 5.61	Pre	138	Primary healthcare center	WAST-Short, AAS	Psychological 19.3%
(Zheng et al., 2020)	China	July–October 2019	CSS	813	Mean 28.98 ± 4.52	Pre	n.a.	Community-based	AAS	Psychological 11.07%
(Zou et al., 2015)	China	October 2006–February 2007	CSS	223 (86 in DV group, 137 in non-DV group)	DV group: 27.8 ± 2.7; non-DV group: 27.2 ± 3.0	Pre	23	Primary healthcare center	AAS	Psychological 63.9%; psychological + sexual 33.7%; psychological + sexual + physical 2.3%

AAS: Abuse Assessment Screen; ANC: antenatal care; CAS: Composite Abuse Scale; CDC BRFSS: Centers for Disease Control–Behavioral Risk Factor Surveillance System; CCS: case–control study; CSS: cross-sectional study; CTS: Conflict Tactics Scale; CTS2: Revised Conflict Tactics Scale; DV: domestic violence; DVWDS: Domestic Violence During Women’s Different Stages; HITS: Hurt, Insult, Threaten, Scream; IPV: intimate partner violence; IQR: Interquartile Range; ISA: Index of Spouse Abuse; MMS: mixed-methods study; NorAQ: Norwegian Abuse Questionnaire; NVAWS: National Violence Against Women Survey; PCS: prospective cohort study; PMWI: Psychological Maltreatment of Women Inventory; Pre: pregnancy; Pue: puerperium; QI: quality improvement pilot study; RCT: randomized controlled trial; SD: Standard Deviation; TOP: termination of pregnancy; USA: United States of America; VAWI: Violence Against Women Instrument; WAST: Woman Abuse Screening Tool; WAST-Short: Woman Abuse Screening Tool—Short Version; WEB: Women’s Experience with Battering; WHO-WHLEQ: World Health Organization—Women’s Health and Life Experiences Questionnaire.

#### 3.2.3. Sexual IPV

A total of 63 studies assessing sexual abuse during pregnancy were included in this systematic review. Study-level details, including year, country, study design, sample size, age, women’s status, setting, and used tools, are summarized in Table 4.

These studies were published between 2013 and 2023 and carried out in 34 countries across different regions, including Africa (Ethiopia, Uganda, Nigeria, South Africa, Tanzania, Egypt, Kenya, Namibia, Zimbabwe), Asia (Saudi Arabia, Iran, Nepal, Bangladesh, India, Pakistan, Malaysia, Vietnam, China), Europe (Sweden, Belgium, Denmark, Estonia, Greece, Iceland, United Kingdom, Norway, Portugal, Spain), South America (Brazil), Oceania (Australia, Vanuatu), and North America (USA). Regarding study design, most of them were a CSS (n = 53), followed by PCS (n = 4), CCS (n = 2), RCT (n = 2), MMS (n = 1), and QI (n = 1). The sample sizes varied considerably, ranging from 65 to 7174 participants. The ages of participants ranged from 13 to 50 years. N = 54 studies focused on pregnant women, n = 5 on postpartum women, and 4 included both pregnancy and postpartum women. The data were collected in various healthcare and community settings, with most studies conducted in hospital-based environments (n = 44), 13 of which were conducted in primary healthcare centers, 5 in community-based settings, and 1 using a mobile app. Prevalence estimates for sexual violence showed considerable variability, ranging from 0% to 45%. Sexual violence among pregnant and postpartum women was assessed using various validated instruments; as in the case of physical violence, the most frequently employed tool was the WHO-WHLEQ (n = 21).

The fixed-effects model yielded an ES of 0.16 (95% CI: 0.15–0.16, *p* < 0.001), based on data from 44,284 participants. Nevertheless, substantial heterogeneity was detected (I^2^ = 98.15%, *p* < 0.001). When the random-effects model was applied, the estimated event rate decreased to 0.09 (95% CI: 0.07–0.11, *p* < 0.001). Evidence of publication bias emerged from visual inspection of the funnel plot and was further supported by Egger’s regression test (intercept = −6.79, *p* < 0.01). These results are presented in Figure S3 (a: forest plot; b: funnel plot) and Table S2.

**Table 4:** Characteristics of studies assessing sexual IPV (n = 63), extracted from the total 98 included articles.

Author, Year	Country	Study Period	Study Design	Sample Size	Age in Years (Range or Mean and SD)	Woman Status	People Lost (Attrition Rate)	Setting	Tool Used to Assess the Outcome	Women Victims of Sexual Violence (Prevalence)
(Abebe Abate et al., 2016)	Ethiopia	April 2014	CSS	282	Mean 27 ± 6.1; range 15–44	Pre	17	Community-based	WHO-WHLEQ	Sexual 30.2%
(Abujilban et al., 2022)	Jordan	September–December 2014	CSS	247	Mean 27.3 ± 5.9	Pre and Pue	n.a.	Hospital-based	WHO-WHLEQ	Sexual 8.9%
(Almeida et al., 2017)	Portugal	February–June 2012	CSS	852	Mean 30.69 ± 5.54; range 18–44	Pre	352	Hospital-based	WHO-WHLEQ	Sexual 19.6%
(Antoniou & Iatrakis, 2019)	Greece	August–September 2009	CSS	546	Mean 32.95 ± 6.78	Pre	n.a.	Hospital-based	AAS	Sexual 1.9%
(Asiimwe et al., 2022)	Uganda	October 2018–February 2019	CSS	100	Mean 17.8 ± 1.26)	Pre and Pue	n.a.	Hospital-based	VAWI	Sexual 45.0%
(Atilla et al., 2023)	Turkey	September–October 2021	CSS	456	Mean 26.66 ± 5.45	Pre	24	Hospital-based	IPV During Pregnancy Questionnaire	Sexual 5.7%
(Avcı et al., 2023)	Turkey	October 2017–August 2018	CSS	255	Mean 28.57 ± 6.17	Pre	n.a.	Primary healthcare center	DVWDS	Sexual 7.4%
(Baǧcioǧlu et al., 2014)	Turkey	n.a.	CSS	317	Mean 27.4 ± 5.9	Pre	2	Hospital-based	AAS	Sexual 5.0%
(Bahrami-Vazir et al., 2020)	Iran	2014	CSS	525	Mean 25.8 ± 5.1	Pre	25	Primary healthcare center	CTS2	Total IPV 67.0%, of which sexual 30.0%
(Belay et al., 2019)	Ethiopia	February–August 2017	CSS	589	Mean 25; range 16–45	Pre	n.a.	Community-based	WHO-WHLEQ	Sexual 9.5%
(Bernstein et al., 2016)	South Africa	March 2013–April 2014	CSS	623	Median age 28; range 18–44	Pre	n.a.	Primary healthcare center	VAWI	Sexual 2.0%
(Bikinesi et al., 2017)	Namibia	n.a.	CSS	386	Mean 27.5 ± 6.8	Pre	n.a.	Primary healthcare center	WHO-WHLEQ	Sexual 1.6%
(L. H. M. de Lima et al., 2016)	Brazil	May 2009–April 2010	CSS	359 (179 adolescents, 180 adults)	Adolescents: mean 17.5 ± 1.4; Adults: mean 26.8 ± 5.8	Pue	8	Hospital-based	AAS	Sexual 1.1%
(Dinmohammadi et al., 2021)	Iran	August 2017	RCT	82 (41 intervention, 41 control)	Mean 27.55 ± 5.13 (intervention), 27.26 ± 4.46 (control)	Pre	8	Primary healthcare center	CTS2	Sexual before 27.0% → after 15.0%
(Elkhateeb et al., 2021)	Egypt	n.a.	CSS	513	n.a.	Pre	37	Hospital-based	AAS	Sexual 20.0%
(Farrokh-Eslamlou et al., 2014)	Iran	February–September 2012	CSS	313	Mean 27.9 ± 5.8; range 17–46	Pre	37	Hospital-based	AAS	Sexual 17.2%
(Fekadu et al., 2018)	Ethiopia	March–May 2016	CSS	450	Mean 27 ± 4.5	Pre	n.a.	Hospital-based	WHO-WHLEQ	Sexual 7.6%
(Ferdos et al., 2018)	Bangladesh	July 2015 to April 2016	CSS	443	<20 y 18.5%; 20–24 y 43.9%; 25–35 y 37.6%	Pue	43	Hospital-based	CTS	Sexual 26.3%
(Field et al., 2018)	South Africa	November 2011–August 2012	MMS	376, 95 case notes analyzed qualitatively	Age categories: 18–24 years (39%), 25–29 years (30%), >29 years (31%)	Pre	186	Hospital-based	CTS2	Sexual 26.0%
(Fonseca-Machado et al., 2015)	Brazil	May 2012–May 2013	CSS	358	Mean 25.0 ± 6.3; range 15–43	Pre	n.a.	Hospital-based	WHO-WHLEQ	Sexual 1.6%
(Gharacheh et al., 2015)	Iran	July–December 2012	CSS	328	Abused: mean 26.25 ± 4.12); Non-abused: mean 27.14 ± 4.29)	Pue	13	Primary healthcare center	AAS	Sexual 34.9%
(Gul et al., 2013)	Pakistan	April 2010–March 2011	CSS	129	Mean 31.42 ± 7.02; range 15–50	Pre	n.a.	Hospital-based	AAS	Sexual 20.4%
(Ilori et al., 2023)	Nigeria	March–September 2019	CSS	240	Mean 30.7 ± 5.5	Pre	n.a.	Hospital-based	CAS	Sexual 38.1%
(Islam et al., 2021)	Bangladesh	October 2015–January 2016	CSS	426	Mean 26.28 ± 5.87; range 15–49	Pue	27	Primary healthcare center	WHO-WHLEQ	Sexual 18.5%
(Iyengar et al., 2021)	United Kingdom	3 months in 2016	CSS	120	Mean 25.22 ± 4.93	Pre	n.a.	Hospital-based	WHO-WHLEQ	Sexual + physical 57.0%
(Kana et al., 2020)	Nigeria	January 2017–April 2019	CSS	293	Mean 28.8 ± 5.9 in IPV-exposed group, 29.2 ± 5.7 in unexposed group	Pre	35	Hospital-based	CTS	Sexual 30.7%
(Khaironisak et al., 2017)	Malaysia	March–August 2015	CSS	1200	Mean 29.07 ± 5.39	Pre	n.a.	Hospital-based	WHO-WHLEQ	Sexual 9.8%
(Khatlani et al., 2023)	Pakistan	February–May 2014	CCS	795 women (256 cases with stillbirths, 539 controls with live births)	Mean 29.6 ± 5.9 in stillbirth group; mean 28.7 ± 5.7 in live birth group	Pre	n.a.	Community-based	WHO-WHLEQ	Sexual 9.81%
(Koirala, 2022)	Nepal	June–September 2020	CSS	220	Mean 30.18 ± 5.70	Pre	n.a.	Hospital-based	VAWI	Sexual 22.7%
(Krishnamurti et al., 2021)	USA	January–May 2020	QI	959 (552 before shelter-in-place, 407 during shelter-in-place)	n.a.	Pre	n.a.	Mobile app	CDC BRFSS (for physical and sexual IPV), WEB (for psychological IPV)	Sexual before 0.4% (552), during: 0.2% (407)
(L. da S. Lima et al., 2020)	Brazil	September–October 2018	CSS	65	Mean 23.88; range 15–42	Pre	n.a.	Primary healthcare center	WHO-WHLEQ	Sexual 3.1%
(Luhumyo et al., 2020)	Kenya	April–June 2017	CSS	369	Median age 25 (IQR: 21–31)	Pre	n.a.	Hospital-based	VAWI	Sexual 13.0%
(Lukasse et al., 2014)	Belgium, Iceland, Denmark, Estonia, Norway, Sweden	March 2008–August 2010	PCS	7174	n.a.	Pre	n.a.	Hospital-based	NorAQ	Sexual 0.4%
(Mahenge et al., 2013)	Tanzania	December 2011–April 2012	CSS	1180	Mean 29.0; range 17–43	Pre	20	Hospital-based	CTS	Sexual 20.0%
(McKelvie et al., 2021)	Vanuatu	May–July 2019	CSS	188	Mean 25.7 ± 5.4	Pre	4	Hospital-based	VAWI	Sexual 7.4%
(Mohamed et al., 2013)	Saudi Arabia	October 2012–February 2013	CSS	404	Mean 31.19 ± 7.36	Pre	12	Primary healthcare center	WAST	Sexual 14.0%
(Musa et al., 2020)	Ethiopia	November 2018–April 2019	CSS	648	n.a.	Pre	n.a.	Hospital-based	WHO-WHLEQ	Sexual 3.7%
(Naghizadeh et al., 2021)	Iran	May–August 2020	CSS	250	Mean 30.57 ± 5.87	Pre	n.a.	Hospital-based	WHO-WHLEQ	Sexual 12.4%
(Nhi et al., 2019)	Vietnam	May 2014–August 2015	PCS	1274	Mean 26; range 16–46	Pre and Pue	63	Hospital-based	WHO-WHLEQ	Sexual 9.8%
(Njoku et al., 2021)	Nigeria	January–March 2017	CSS	400	Mean 30.1 ± 2.47; range 20–45	Pre	n.a.	Hospital-based	AAS	Sexual 7.3%%
(Okunola et al., 2021)	Nigeria	March 2019 and September 2019	PCS	363	Mean 30 ± 5.3	Pre	0	Hospital-based	Ongoing abuse screen	Sexual 3.6%
(Omoronyia et al., 2020)	Nigeria	n.a.	CSS	250	29.7 ± 6.1	Pre	n.a.	Hospital-based	CAS	Sexual 29.2%; psychological + sexual 10.8%
(Pun et al., 2019)	Nepal	June 2015–September 2016	PCS	1381	Age categories: 15–19 (5.6%), 20–24 (42.8%), 25–29 (37.6%), ≥30 (14.0%)	Pre	623	Hospital-based	VAWI	Sexual 0.9%
(Pun et al., 2018)	Nepal	November 2014–November 2015	CSS	1011	Mean 24.4 ± 4.0	Pre	28	Hospital-based	AAS	Sexual 1.6%
(Rasch et al., 2018)	Tanzania and Vietnam	n.a.	CSS	2425 (1116 in Tanzania, 1309 in Vietnam)	n.a.	Pre	n.a.	Hospital-based	VAWI	Tanzania: sexual 15.4%; Vietnam: sexual 9.9%
(M. R. C. Ribeiro et al., 2017)	Brazil	February 2010–June 2011	CSS	1446 (São Luís), 1378 (Ribeirão Preto)	n.a.	Pre	1	Primary healthcare center	VAWI	Sexual 2.8%
(Samal & Poornesh, 2022)	India	October–November 2016	CSS	200	Range 19–40	Pre	n.a.	Hospital-based	AAS	Sexual 0%
(Sapkota et al., 2021)	Nepal	June–August 2018	RCT	140	Mean 25.3 ± 5.4	Pre	3	Hospital-based	AAS	Sexual 15.0%
(Shamu et al., 2014)	Zimbabwe	May–September 2011	CSS	1951	n.a.	Pre and Pue	n.a.	Hospital-based	WHO-WHLEQ	Sexual 22.6%
(Shamu et al., 2013)	Zimbabwe	May–September 2011	CSS	2042	Mean 26 ± 5.71; range 15–48	Pre	59	Primary healthcare center	WHO-WHLEQ	Sexual 38.9%; sexual and/or physical 46.2%
(Shannon et al., 2016)	USA	August 2005–October 2007	CSS	77	Mean 24.96 ± 3.83	Pre	n.a.	Hospital-based	NVAWS, CTS2, PMWI	Sexual 14.3%
(Shrestha et al., 2016)	Nepal	September–November 2015	CSS	404	Mean 25.5 ± 4.3; 43.8% <25	Pre	n.a.	Hospital-based	WHO-WHLEQ	Sexual 17.3%
(Silva et al., 2022)	Brazil	August–October 2017	CSS	327	Not explicitly reported; categorized as ≤40 years and >40 years	Pre	n.a.	Hospital-based	VAWI	Sexual 3.08%
(Silva & Leite, 2019)	Brazil	August–October 2017	CSS	330	Not explicitly reported; categorized as 14–19 years and ≥20 years	Pre	n.a.	Hospital-based	VAWI	Sexual 2.7%
(Sobhani et al., 2018)	Iran	September–December 2014	CSS	402	Mean 28.24 ± 5.91; range 13–44	Pre	n.a.	Hospital-based	WHO-WHLEQ	Sexual 16.7%
(Takelle et al., 2023)	Ethiopia	May–June 2022	CSS	473	Mean 28.18 ± 5.28; range 18–41	Pre	12	Hospital-based	WHO-WHLEQ	Sexual 3.2%
(Utaile et al., 2023)	Ethiopia	July–October 2020	CSS	1535	Mean 26.3 ± 4.7	Pre	n.a.	Community-based	WHO-WHLEQ	Sexual 19.3%
(Velasco et al., 2014)	Spain	2009	CSS	779	Mean 29.9 ± 5.6	Pre	n.a.	Hospital-based	AAS, ISA	AAS sexual 0.5%
(Wangel et al., 2016)	Sweden	March–November 2008	CSS	1003	Age groups: <25 years (11.2%), 25–29 (32%), 30–35 (43.2%), >35 (13.7%)	Pre	22	Hospital-based	NorAQ	Sexual 15.5%
(Watson & Taft, 2013)	Australia	April 2002–March 2004	CCS	1726	n.a.	Pre	54	Hospital-based	CAS	Sexual 5.1%
(Zapata-Calvente et al., 2022)	Spain	January 2017–March 2019	CSS	592	Mean 31.82 ± 5.61	Pre	138	Primary healthcare center	WAST-Short, AAS	Sexual 2.4%
(Zheng et al., 2020)	China	July–October 2019	CSS	813	Mean 28.98 ± 4.52	Pre	n.a.	Community-based	AAS	Sexual 0.86%
(Zou et al., 2015)	China	October 2006–February 2007	CSS	223 (86 in DV group, 137 in non-DV group)	DV group: 27.8 ± 2.7; non-DV group: 27.2 ± 3.0	Pre	23	Primary healthcare center	AAS	Sexual + psychological 33.7%; Sexual + physical + psychological 2.3%

AAS: Abuse Assessment Screen; CAS: Composite Abuse Scale; CDC BRFSS: Centers for Disease Control—Behavioral Risk Factor Surveillance System; CCS: case–control study; CSS: cross-sectional study; CTS: Conflict Tactics Scale; CTS2: Revised Conflict Tactics Scale; DV: domestic violence; DVWDS: Domestic Violence During Women’s Different Stages; IPV: intimate partner violence; IQR: Interquartile Range; MMS: mixed-methods study; NorAQ: Norwegian Abuse Questionnaire; NVAWS: National Violence Against Women Survey; PCS: prospective cohort study; PMWI: Psychological Maltreatment of Women Inventory; Pre: pregnancy; Pue: puerperium; QI: quality improvement pilot study; RCT: randomized controlled trial; SD: Standard Deviation; USA: United States of America; VAWI: Violence Against Women Instrument; WAST: Woman Abuse Screening Tool; WAST-Short: Woman Abuse Screening Tool—Short Version; WEB: Women’s Experience with Battering; WHO-WHLEQ: World Health Organization—Women’s Health and Life Experiences Questionnaire.

#### 3.2.4. Any IPV

Some of the included articles (n = 71) report the prevalence of violence during pregnancy or the postpartum period in a generic way, without specifying its typology; we refer to these cases as “any IPV”. However, this does not mean that these articles do not also include separate data on individual forms of violence (physical, psychological, etc.). Study-level details, including year, country, study design, sample size, age, women’s status, setting, and used tools, are summarized in Table 5.

These articles were published between 2013 and 2023 and conducted across 30 countries spanning several global regions: Africa (Ethiopia, Nigeria, Egypt, Kenya, Namibia, South Africa, Tanzania, Zimbabwe), Asia (China, India, Iran, Japan, Jordan, Malaysia, Nepal, Saudi Arabia, South Korea, Thailand, Vietnam), Europe (Denmark, United Kingdom, Greece, Portugal, Spain), North America (United States), South America (Brazil, Peru), and Oceania (Australia, Vanuatu). The predominant study design was CSS (n = 56), followed by PCS (n = 10); in addition, each of the other study design categories present in the 98 included studies is represented by a single article: RCS, RCT, MMS, CCS, and qualitative study (QUAL). The studies included a wide range of sample sizes, from 43 to 16,068 participants, and participants’ ages ranged from 13 to 48 years. Most studies focused exclusively on pregnant women (n = 56), while others included postpartum women (n = 6) or both groups (n = 9). Prevalence estimates for any IPV showed considerable variability, ranging from 3.5% to 93.1%. Some studies provided stratified data: one study found IPV in 48.3% of women at follow-up, while 51.7% remained exposed to IPV; differences between geographical locations were also observed, with one study reporting an 8.53% prevalence in Denmark and 17.03% in Spain, and another showing higher rates in rural (40.56%) compared to urban (37.25%) settings. Data were collected primarily in hospital-based environments (n = 51), and others were obtained in primary care centers (n = 13) and community settings (n = 7). Various validated tools were used across studies to assess any form of IPV among pregnant and postpartum women. The most frequently employed instrument was the WHO-WHLEQ (n = 19), followed closely by the Abuse Assessment Screen (AAS) (n = 18).

In the meta-analysis, the fixed-effects model produced an estimated event rate of 0.28 (95% CI: 0.27–0.28, *p* < 0.001), based on data from 70,860 participants. However, considerable heterogeneity was observed (I^2^ = 99.21%, *p* < 0.001). Under the random-effects model, the estimated event rate slightly decreased to 0.26 (95% CI: 0.22–0.30, *p* < 0.001). No evidence of publication bias was detected, as confirmed by both the symmetrical funnel plot and Egger’s regression test (intercept = 0.46, *p* = 0.860). These findings are shown in Figure S5 (a: forest plot; b: funnel plot) and Table S2.

**Table 5:** Characteristics of studies assessing any IPV (n = 71), extracted from the total 98 included articles.

Author, Year	Country	Study Period	Study Design	Sample Size	Age in Years (Range or Mean and SD)	Woman Status	People Lost (Attrition Rate)	Setting	Tool Used to Assess the Outcome	Women Victims of any IPV (Prevalence)
(Abebe Abate et al., 2016)	Ethiopia	April 2014	CSS	282	Mean 27 ± 6.1; range 15–44	Pre	17	Community-based	WHO-WHLEQ	Any IPV 44.5%
(Abujilban et al., 2022)	Jordan	September–December 2014	CSS	247	Mean 27.3 ± 5.9	Pre and Pue	n.a.	Hospital-based	WHO-WHLEQ	Any IPV 93.1%
(Alhalal et al., 2022)	Saudi Arabia	December 2019–March 2020	CSS	684	Mean 31.19 ± 7.36	Pre and Pue	66	Hospital-based	CAS	Any IPV 28.9%
(Alhusen et al., 2015)	USA	February 2009–March 2010	CSS	166	Mean 23.3 ± 5.4	Pre	n.a.	Hospital-based	AAS	Any IPV 19.3%
(Almeida et al., 2017)	Portugal	February–June 2012	CSS	852	Mean 30.69 ± 5.54; range 18–44	Pre	352	Hospital-based	WHO-WHLEQ	Any IPV 43.4%
(Andreasen et al., 2023)	Denmark, Spain	2021–2022	PCS	Total 16,068 (Denmark 14,013, Spain 2055)	Mean Denmark: 28.7 ± 5.1; Spain: 31.6 ± 5.9	Pre	77	Hospital-based	AAS	Any IPV 9.62%: Denmark 8.53%; Spain 17.03%
(Antoniou & Iatrakis, 2019)	Greece	August–September 2009	CSS	546	Mean 32.95 ± 6.78	Pre	n.a.	Hospital-based	AAS	Any IPV 6.0%
(Gómez Aristizábal et al., 2022)	Brazil	February 2010 and June 2011	PCS	1447	Mean 26.1 ± 5.4	Pre	317	Primary healthcare center	VAWI	Any IPV 49.7%
(Atilla et al., 2023)	Turkey	September–October 2021	CSS	456	Mean 26.66 ± 5.45	Pre	24	Hospital-based	IPV During Pregnancy Questionnaire	Any IPV 44.1%
(Avcı et al., 2023)	Turkey	October 2017–August 2018	CSS	255	Mean 28.57 ± 6.17	Pre	n.a.	Primary healthcare center	DVWDS	Any IPV 9.8%
(Baǧcioǧlu et al., 2014)	Turkey	n.a.	CSS	317	Mean 27.4 ± 5.9	Pre	2	Hospital-based	AAS	Any IPV 10.3%
(Belay et al., 2019)	Ethiopia	February–August 2017	CSS	589	Mean 25; range 16–45	Pre	n.a.	Community-based	WHO-WHLEQ	Any IPV 21.2%
(Bernstein et al., 2016)	South Africa	March 2013–April 2014	CSS	623	Median age 28; range 18–44	Pre	n.a.	Primary healthcare center	VAWI	Any IPV 21%
(Bikinesi et al., 2017)	Namibia	n.a.	CSS	386	Mean 27.5 ± 6.8	Pre	n.a.	Primary healthcare center	WHO-WHLEQ	Any IPV 8.0%
(Boonnate et al., 2015)	Thailand	n.a.	CSS	230	Mean 28.98 ± 5.17	Pre	n.a.	Hospital-based	ISA	Any IPV 11.7%
(Caprara et al., 2020)	Brazil	2011–2016	PCS	232	Mean 27.4 ± 6.7	Pue	n.a.	Hospital-based	AAS	Any IPV 15.1%
(Chen et al., 2017)	USA	January 2003–December 2009	RCS	1438	Mean 26.0 (victims: 27.1; non-victims: 25.9)	Pre	n.a.	Hospital-based	HITS	Any IPV 7.5%
(Dinmohammadi et al., 2021)	Iran	August 2017	RCT	82 (41 intervention, 41 control)	Mean 27.55 ± 5.13 (intervention), 27.26 ± 4.46 (control)	Pre	8	Primary healthcare center	CTS2	Any IPV before 59.0% → after 38.0%
(Elkhateeb et al., 2021)	Egypt	n.a.	CSS	513	n.a.	Pre	37	Hospital-based	AAS	Any IPV 50.8%
(Farrokh-Eslamlou et al., 2014)	Iran	February–September 2012	CSS	313	Mean 27.9 ± 5.8; range 17–46	Pre	37	Hospital-based	AAS	Any IPV 55.9%
(Fekadu et al., 2018)	Ethiopia	March–May 2016	CSS	450	Mean 27 ± 4.5	Pre	n.a.	Hospital-based	WHO-WHLEQ	Any IPV 58.7%
(Field et al., 2018)	South Africa	November 2011–August 2012	MMS	376, 95 case notes analyzed qualitatively	Age categories: 18–24 years (39%), 25–29 years (30%), >29 years (31%)	Pre	186	Hospital-based	CTS2	Any IPV 15.0%
(Fisher et al., 2013)	Vietnam	December 2009–June 2011	PCS	417 (pregnancy), 453 (postpartum)	Mean 26.1 ± 4.8	Pre and Pue	80	Community-based	WHO-WHLEQ	Any IPV 3.8% (pregnancy); any IPV 5.9% (postpartum)
(Fonseca-Machado et al., 2015)	Brazil	May 2012–May 2013	CSS	358	Mean 25.0 ± 6.3; range 15–43	Pre	n.a.	Hospital-based	WHO-WHLEQ	Any IPV 17.6%
(Gashaw et al., 2019)	Ethiopia	November 2015–March 2016	CSS	720	Mean 25 ± 5.0	Pre	n.a.	Hospital-based	AAS	Any IPV 44.0%
(Gebrekristos et al., 2023)	South Africa	July 2017–April 2018	CSS	90	Mean 17.5 ± 1.4; range 14–19	Pre and Pue	29	Hospital-based	CTS	Any IPV 40.0%
(Gelaye et al., 2016)	Peru	February 2012–March 2014	CSS	2970	Mean 28.1 ± 6.3; range 18–35	Pre	89	Hospital-based	WHO-WHLEQ	Any IPV 36.7%
(Gharacheh et al., 2015)	Iran	July–December 2012	CSS	328	Abused: mean 26.25 ± 4.12); non-abused: mean 27.14 ± 4.29)	Pue	13	Primary healthcare center	AAS	Any IPV 44.5%
(Hooker & Taft, 2021)	Australia	2011	CSS	2621	Mean 34.0	Pue	n.a.	Primary healthcare center	CAS	Any IPV 6.8%
(Ilori et al., 2023)	Nigeria	March–September 2019	CSS	240	Mean 30.7 ± 5.5	Pre	n.a.	Hospital-based	CAS	Any IPV 45.8%
(Iyengar et al., 2021)	United Kingdom	3 months in 2016	CSS	120	Mean 25.22 ± 4.93	Pre	n.a.	Hospital-based	WHO-WHLEQ	Any IPV 35.0%
(Kana et al., 2020)	Nigeria	January 2017–April 2019	CSS	293	Mean 28.8 ± 5.9 in IPV-exposed group, 29.2 ± 5.7 in unexposed group	Pre	35	Hospital-based	CTS	Any IPV 66.6%
(Kataoka & Imazeki, 2018)	Japan	September–December 2011	QUAL	43	Age categories: <20 years (2.3%), 20–29 (32.6%), ≥30 (65.1%)	Pue	5	Hospital-based	VAWS	Any IPV 18.6%
(Khaironisak et al., 2017)	Malaysia	March–August 2015	CSS	1200	Mean 29.07 ± 5.39	Pre	n.a.	Hospital-based	WHO-WHLEQ	Any IPV 35.9%
(Kian et al., 2019)	Iran	2015–2016	CSS	400 (200 rural, 200 urban)	Mean 29.15 ± 5.37 (urban), 28.25 ± 6.3 (rural)	Pre and Pue	n.a.	Primary healthcare center	Standardized violence questionnaire	Any IPV: rural 40.56%; urban 37.25%
(Kita et al., 2017)	Japan	July 2013–July 2014	PCS	453	Mean 32.1 ± 4.9; range 19–46	Pre	502	Hospital-based	WAST-Short	Any IPV 12.1%
(Koirala, 2022)	Nepal	June–September 2020	CSS	220	Mean 30.18 ± 5.70	Pre	n.a.	Hospital-based	VAWI	Any IPV 32.7%
(Lee et al., 2023)	South Korea	2020–2021	CSS	5616	Range 16–48	Pre and Pue	337	Primary healthcare center	HITS	Any IPV 7.6%
(Luhumyo et al., 2020)	Kenya	April–June 2017	CSS	369	Median age 25 (IQR: 21–31)	Pre	n.a.	Hospital-based	VAWI	Any IPV 34.1%
(Mahenge et al., 2013)	Tanzania	December 2011–April 2012	CSS	1180	Mean 29.0; range 17–43	Pre	20	Hospital-based	CTS	Any IPV 27.0%
(Stella Martin-de-las-Heras et al., 2015)	Spain	n.a.	CSS	779	Mean 29.9 ± 5.6	Pre	153	Hospital-based	ISA	Any IPV 21.3%
(McKelvie et al., 2021)	Vanuatu	May–July 2019	CSS	188	Mean 25.7 ± 5.4	Pre	4	Hospital-based	VAWI	Any IPV 44.68%
(Mohamed et al., 2013)	Saudi Arabia	October 2012–February 2013	CSS	404	Mean 31.19 ± 7.36	Pre	12	Primary healthcare center	WAST	Any IPV 52.0%
(Musa et al., 2020)	Ethiopia	November 2018–April 2019	CSS	648	n.a.	Pre	n.a.	Hospital-based	WHO-WHLEQ	Any IPV 39.81%
(Naghizadeh et al., 2021)	Iran	May–August 2020	CSS	250	Mean 30.57 ± 5.87	Pre	n.a.	Hospital-based	WHO-WHLEQ	Any IPV 35.2%
(Nhi et al., 2019)	Vietnam	May 2014–August 2015	PCS	1274	Mean 26; range 16–46	Pre and Pue	63	Hospital-based	WHO-WHLEQ	Any IPV 35.3%
(Njoku et al., 2021)	Nigeria	January–March 2017	CSS	400	Mean 30.1 ± 2.47; range 20–45	Pre	n.a.	Hospital-based	AAS	Any IPV 43.0%
(Nongrum et al., 2014)	India	Not reported	PCS	150	Mean 26.32 ± 4.22	Pre	18	Hospital-based	AAS	Any IPV 7.3%
(Okunola et al., 2021)	Nigeria	March 2019 and September 2019	PCS	363	Mean 30 ± 5.3	Pre	0	Hospital-based	Ongoing abuse screen	Any IPV 15.4%
(Omoronyia et al., 2020)	Nigeria	n.a.	CSS	250	29.7 ± 6.1	Pre	n.a.	Hospital-based	CAS	Any IPV 54.8%
(Priya et al., 2019)	India	December 2013–February 2015	CSS	165	23.8 ± 3.8	Pre	n.a.	Community-based	HITS	Any IPV 23.0%
(Pun et al., 2019)	Nepal	June 2015–September 2016	PCS	1381	Age categories: 15–19 (5.6%), 20–24 (42.8%), 25–29 (37.6%), ≥30 (14.0%)	Pre	623	Hospital-based	VAWI	Any IPV 20.5%
(Pun et al., 2018)	Nepal	November 2014–November 2015	CSS	1011	Mean 24.4 ± 4.0	Pre	28	Hospital-based	AAS	Any IPV 23.7%
(Rasch et al., 2018)	Tanzania and Vietnam	n.a.	CSS	2425 (1116 in Tanzania, 1309 in Vietnam)	n.a.	Pre	n.a.	Hospital-based	VAWI	Tanzania: any IPV 30.2%; Vietnam: any IPV 35.2%
(M. R. C. Ribeiro et al., 2017)	Brazil	February 2010–June 2011	CSS	1446 (São Luís), 1378 (Ribeirão Preto)	n.a.	Pre	1	Primary healthcare center	VAWI	Any IPV 49.6%
(Rishal et al., 2020)	Nepal	August 2014–November 2016	PCS	1010 screened, 181 reported IPV	n.a.	Pre	119	Hospital-based	AAS	Any IPV 17.9%; at follow-up: no longer IPV 48.3%; still IPV 51.7%
(Samal & Poornesh, 2022)	India	October–November 2016	CSS	200	Range 19–40	Pre	n.a.	Hospital-based	AAS	Any IPV 6.5%
(Sánchez et al., 2023)	Brazil	July 2019–September 2021	CSS	600	Mean 27.0 ± 8.58; range 13–47	Pre and Pue	n.a.	Hospital-based	AAS, WAST, HITS	WAST: Any IPV 6.7%; HITS: Any IPV 3.5%
(Shamu et al., 2014)	Zimbabwe	May–September 2011	CSS	1951	n.a.	Pre and Pue	n.a.	Hospital-based	WHO-WHLEQ	Any IPV 32.8%
(Shamu et al., 2013)	Zimbabwe	May–September 2011	CSS	2042	Mean 26 ± 5.71; range 15–48	Pre	59	Primary healthcare center	WHO-WHLEQ	Any IPV 63.1%
(Shannon et al., 2016)	USA	August 2005–October 2007	CSS	77	Mean 24.96 ± 3.83	Pre	n.a.	Hospital-based	NVAWS, CTS2, PMWI	Any IPV 75.3%
(Shrestha et al., 2016)	Nepal	September–November 2015	CSS	404	Mean 25.5 ± 4.3; 43.8% <25	Pre	n.a.	Hospital-based	WHO-WHLEQ	Any IPV 27.2%
(Sobhani et al., 2018)	Iran	September–December 2014	CSS	402	Mean 28.24 ± 5.91; range 13–44	Pre	n.a.	Hospital-based	WHO-WHLEQ	Any IPV 48.5%
(Sulaiman et al., 2021)	Nigeria	November 2018–August 2019	CSS	403	Mean 33 ± 4.9	Pre	8	Hospital-based	HITS	Any IPV 56.3%
(Sussmann et al., 2020)	Brazil	January 2006–March 2007	CSS	700	Age categories: 16–20 years, 21–30 years, >31 years	Pue	128	Community-based	VAWI	Any IPV 24.7%
(Suzuki et al., 2018)	Japan	April–October 2016	CSS	470	n.a.	Pre	2	Hospital-based	VAWS	Any IPV 4.1%
(Utaile et al., 2023)	Ethiopia	July–October 2020	CSS	1535	Mean 26.3 ± 4.7	Pre	n.a.	Community-based	WHO-WHLEQ	Any IPV 48.0%
(Velasco et al., 2014)	Spain	2009	CSS	779	Mean 29.9 ± 5.6	Pre	n.a.	Hospital-based	AAS, ISA	AAS: Any IPV 7.7%; ISA: Any IPV 21.0%
(Watson & Taft, 2013)	Australia	April 2002–March 2004	CCS	1726	n.a.	Pre	54	Hospital-based	CAS	Any IPV 14.9%
(Zapata-Calvente et al., 2022)	Spain	January 2017–March 2019	CSS	592	Mean 31.82 ± 5.61	Pre	138	Primary healthcare center	WAST-Short, AAS	Any IPV 9.5%
(Zheng et al., 2020)	China	July–October 2019	CSS	813	Mean 28.98 ± 4.52	Pre	n.a.	Community-based	AAS	Any IPV 15.62%

AAS: Abuse Assessment Screen; CAS: Composite Abuse Scale; CCS: case–control study; CSS: cross-sectional study; CTS: Conflict Tactics Scale; CTS2: Revised Conflict Tactics Scale; DVWDS: Domestic Violence During Women’s Different Stages; HITS: Hurt, Insult, Threaten, Scream; IPV: intimate partner violence; IQR: Interquartile Range; ISA: Index of Spouse Abuse; MMS: mixed-methods study; NVAWS: National Violence Against Women Survey; PCS: prospective cohort study; PMWI: Psychological Maltreatment of Women Inventory; Pre: pregnancy; Pue: puerperium; QUAL: qualitative study; RCS: retrospective cohort study; RCT: randomized controlled trial; SD: Standard Deviation; USA: United States of America; VAWI: Violence Against Women Instrument; WAST: Woman Abuse Screening Tool; WAST-Short: Woman Abuse Screening Tool—Short Version; VAWS: Violence Against Women Screen; WHO-WHLEQ: World Health Organization—Women’s Health and Life Experiences Questionnaire.

#### 3.2.5. Verbal IPV

Out of the 98 total included articles, this review identified 8 studies that examined verbal violence during pregnancy. Study-level details, including year, country, study design, sample size, age, women’s status, setting, and used tools, are summarized in Table 6.

These studies were published between 2013 and 2023 across various global regions, including Africa (Egypt, Nigeria), Asia (Pakistan, South Korea, India), and Europe (Turkey, Malta). All included studies employed a CSS design. The participant numbers varied, with sample sizes ranging from 129 to 5616. Participants’ ages spanned from 15 to 50 years. All participants were pregnant women, except in one study that also included the puerperium period. Most studies were conducted in hospital-based settings, with the exception of one study carried out in a primary healthcare center. Reported prevalence rates for verbal violence exhibited a wide range, from 0.3% up to 85.5%. Instruments to assess IPV with a specific focus on verbal abuse included the Abuse Assessment Screen (AAS) (n = 5), the Hurt, Insult, Threaten, Scream (HITS) tool (n = 2), and the WHO Violence Against Women Instrument (VAWI) (n = 1). These tools had either been previously validated in their respective languages and cultural contexts or were adapted versions of internationally recognized IPV screening instruments.

**Figure 2:**
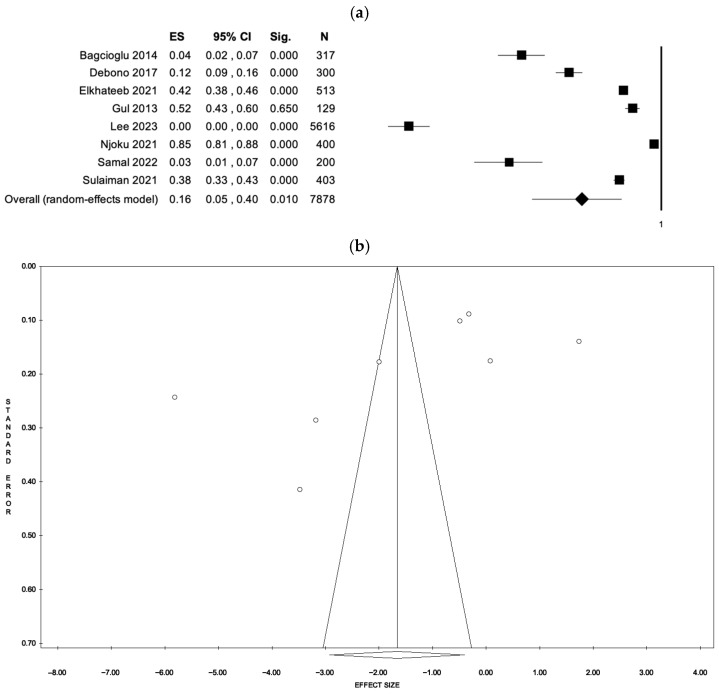
(**a**) A forest plot and (**b**) funnel plot of the random-effects model assessing verbal IPV (Baǧcioǧlu et al., 2014, Debono et al., 2017, Elkhateeb et al., 2021, Gul et al., 2013, Lee et al., 2023, Njoku et al., 2021, Samal & Poornesh, 2022, Sulaiman et al., 2021).

The fixed-effects model estimated an event rate of 0.36 (95% CI: 0.34–0.38, *p* < 0.001), based on data from 7878 participants. Nonetheless, substantial heterogeneity was detected (I^2^ = 99.26%, *p* < 0.001). When applying the random-effects model, the event rate was lower, estimated at 0.16 (95% CI: 0.05–0.40, *p* < 0.001). No evidence of publication bias was found, as suggested by the symmetry of the funnel plot and confirmed by Egger’s regression test (intercept = –15.12, *p* = 0.130). These results are presented in Figure 2 (a: forest plot; b: funnel plot) and Table S2.

#### 3.2.6. Economic IPV

Research on economic violence during pregnancy remains limited, with only seven studies. Study-level details, including year, country, study design, sample size, age, woman status, setting, and used tools, are summarized in Table 7.

These studies were published between 2013 and 2023 and specifically addressed this form of abuse. These studies were carried out in diverse geographical contexts, including Turkey (three studies), Namibia, Pakistan, Nigeria, and India. Despite the limited number, all studies consistently applied a CSS design. The populations examined consisted exclusively of pregnant women, with sample sizes ranging from 129 to 456 and participant ages between 15 and 50 years. While the majority of studies were conducted in hospital-based settings, two were implemented in Primary Healthcare Center contexts. Reported prevalence rates of economic violence varied notably, spanning from 2% to 48.3%. All studies used validated instruments for assessing IPV, including tools such as the AAS (n = 4), the WHO Women’s Health and Life Experiences Questionnaire, the Domestic Violence to Women Determination Scale (Turkey), and the IPV During Pregnancy Questionnaire. These tools allowed for standardized evaluation of economic abuse, alongside other IPV subtypes.

**Figure 3:**
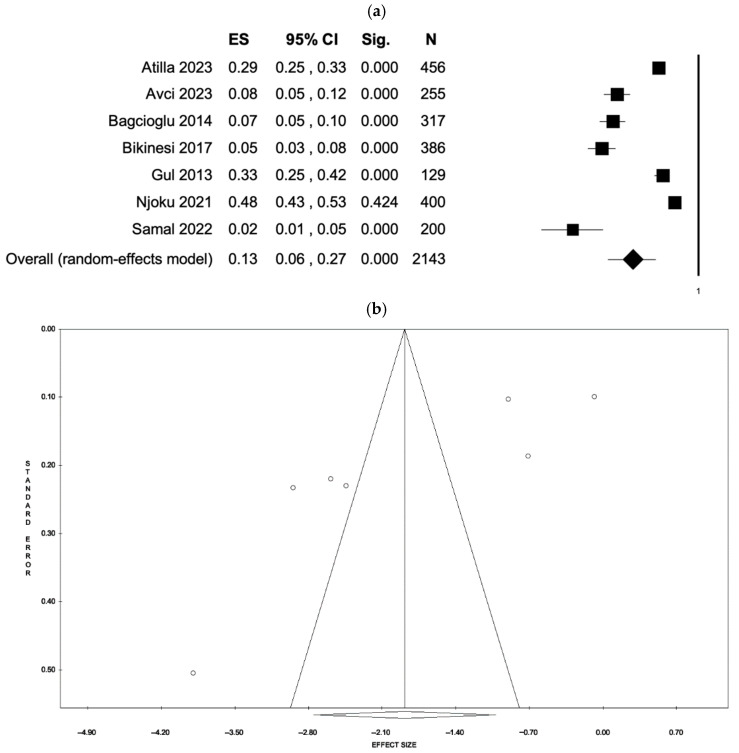
(**a**) A forest plot and (**b**) funnel plot of the random-effects model assessing economic IPV (Atilla et al., 2023, Avcı et al., 2023, Baǧcioǧlu et al., 2014, Bikinesi et al., 2017, Gul et al., 2013, Njoku et al., 2021, Samal & Poornesh, 2022).

The fixed-effects model estimated an event rate of 0.27 (95% CI: 0.25–0.29, *p* < 0.001), based on data from 2143 participants. However, substantial heterogeneity was observed (I^2^ = 97.86%, *p* < 0.001). When the random-effects model was applied, the estimated event rate decreased to 0.13 (95% CI: 0.06–0.27, *p* < 0.001). Evidence of publication bias was identified, as indicated by funnel plot asymmetry and confirmed by Egger’s regression test (intercept = −3.48, *p* = 0.018). These findings are presented in Figure 3 (a: forest plot; b: funnel plot) and Supplementary Table S2.

**Table 6:** Characteristics of studies assessing verbal IPV (n = 8), extracted from the total 98 included articles.

Author, Year	Country	Study Period	Study Design	Sample Size	Age in Years (Range or Mean and SD)	Woman Status	People Lost (Attrition Rate)	Setting	Tool Used to Assess the Outcome	Women Victims of Verbal Violence (Prevalence)
(Baǧcioǧlu et al., 2014)	Turkey	n.a.	CSS	317	Mean 27.4 ± 5.9	Pre	2	Hospital-based	AAS	Verbal 4.4%
(Debono et al., 2017)	Malta	October 2014–January 2015	CSS	300	Mean 30.7; range: 18–43	Pre	80	Hospital-based	VAWI	Verbal 12.0%
(Elkhateeb et al., 2021)	Egypt	n.a.	CSS	513	n.a.	Pre	37	Hospital-based	AAS	Verbal 41.7%
(Gul et al., 2013)	Pakistan	April 2010–March 2011	CSS	129	Mean 31.42 ± 7.02; range 15–50	Pre	n.a.	Hospital-based	AAS	Verbal 51.9%
(Lee et al., 2023)	South Korea	2020–2021	CSS	5616	Range 16–48	Pre and Pue	337	Primary healthcare center	HITS	Verbal 0.3%
(Njoku et al., 2021)	Nigeria	January–March 2017	CSS	400	Mean 30.1 ± 2.47; range 20–45	Pre	n.a.	Hospital-based	AAS	Verbal 85.5%
(Samal & Poornesh, 2022)	India	October–November 2016	CSS	200	Range 19–40	Pre	n.a.	Hospital-based	AAS	Verbal 3.0%
(Sulaiman et al., 2021)	Nigeria	November 2018–August 2019	CSS	403	Mean 33 ± 4.9	Pre	8	Hospital-based	HITS	Verbal 38.4%

AAS: Abuse Assessment Screen; CSS: cross-sectional study; HITS: Hurt, Insult, Threaten, Scream; Pre: pregnancy; Pue: puerperium; SD: Standard Deviation; VAWI: Violence Against Women Instrument.

**Table 7:** Characteristics of studies assessing economic IPV (n = 7), extracted from the total 98 included articles.

Author, Year	Country	Study Period	Study Design	Sample Size	Age in Years (Range or Mean and SD)	Woman Status	People Lost (Attrition Rate)	Setting	Tool Used to Assess the Outcome	Women Victims of Economic Violence (Prevalence)
(Atilla et al., 2023)	Turkey	September–October 2021	CSS	456	Mean 26.66 ± 5.45	Pre	24	Hospital-based	IPV During Pregnancy Questionnaire	Economic 28.9%
(Avcı et al., 2023)	Turkey	October 2017–August 2018	CSS	255	Mean 28.57 ± 6.17	Pre	n.a.	Primary healthcare center	DVWDS	Economic 8.2%
(Baǧcioǧlu et al., 2014)	Turkey	n.a.	CSS	317	Mean 27.4 ± 5.9	Pre	2	Hospital-based	AAS	Economic 6.6%
(Bikinesi et al., 2017)	Namibia	n.a.	CSS	386	Mean 27.5 ± 6.8	Pre	n.a.	Primary healthcare center	WHO-WHLEQ	Economic 5.2%
(Gul et al., 2013)	Pakistan	April 2010–March 2011	CSS	129	Mean 31.42 ± 7.02; range 15–50	Pre	n.a.	Hospital-based	AAS	Economic 33.3%
(Njoku et al., 2021)	Nigeria	January–March 2017	CSS	400	Mean 30.1 ± 2.47; range 20–45	Pre	n.a.	Hospital-based	AAS	Economic 48.3%
(Samal & Poornesh, 2022)	India	October–November 2016	CSS	200	Range 19–40	Pre	n.a.	Hospital-based	AAS	Economic 2.0%

AAS: Abuse Assessment Screen; CSS: cross-sectional study; DVWDS: Domestic Violence During Women’s Different Stages; IPV: intimate partner violence; Pre: pregnancy; SD: Standard Deviation; WHO-WHLEQ: World Health Organization—Women’s Health and Life Experiences Questionnaire.

#### 3.2.7. Non-Physical Violence

A total of three studies specifically assessed non-physical IPV during pregnancy. Study-level details, including year, country, study design, sample size, age, woman status, setting, and used tools, are summarized in Table 8.

All of them focused exclusively on women during pregnancy, with no assessment in the puerperium period. The studies were conducted in two Asian countries—Japan and Thailand—and were published between 2015 and 2017. The included studies adopted either a CSS (n = 1) or PCS (n = 2) design. Sample sizes ranged from 230 to 562 participants. The mean age of participants was consistently late twenties to early thirties. All studies were hospital-based, recruiting pregnant women from antenatal clinics at university or public hospitals. To assess non-physical violence, the studies used established tools: the Index of Spouse Abuse (ISA) (n = 2) and the Woman Abuse Screening Tool-Short (WAST-Short), both of which had been validated for use in the local contexts. These instruments evaluated non-physical components of IPV, including psychological and emotional abuse, social exclusion, intimidation, and controlling behaviors. The prevalence of non-physical IPV during pregnancy ranged from 3.1% to 4.3%.

**Table 8:** Characteristics of studies assessing non-physical IPV (n = 3), extracted from the total 98 included articles.

Author, Year	Country	Study Period	Study Design	Sample Size	Age in Years (Range or Mean and SD)	Woman Status	People Lost (Attrition Rate)	Setting	Tool Used to Assess the Outcome	Women Victims of Violence (Prevalence)
(Boonnate et al., 2015)	Thailand	n.a.	CSS	230	Mean 28.98 ± 5.17	Pre	n.a.	Hospital-based	ISA	Non-physical 4.3%
(Kita et al., 2017)	Japan	July 2013–July 2014	PCS	453	Mean 32.1 ± 4.9; range 19–46	Pre	502	Hospital-based	WAST-Short	Non-physical 3.1%
(Kita et al., 2016)	Japan	July 2013–July 2014	PCS	562	Mean 32.2 ± 4.9; range 19–46	Pre	393	Hospital-based	ISA	Non-physical 3.6%

CSS: Cross-sectional study; ISA: Index of Spouse Abuse; PCS: prospective cohort study; Pre: pregnancy; SD: Standard Deviation; WAST-Short: Woman Abuse Screening Tool—Short Version.

### 3.3. Sensitivity Analyses by WHO Region and Income Level

Sensitivity analyses stratified by WHO region (Table S3) (World Health Organization, n.d.) and World Bank income classification (Table S4) (Metreau et al., 2024) demonstrated substantial variability in the prevalence of physical, psychological, sexual, and any IPV, with consistently high between-study heterogeneity observed across all subgroup analyses.

For physical IPV, the highest pooled prevalence estimates (ER^) were observed in the African Region (0.23; 95% CI: 0.23–0.24), Eastern Mediterranean Region (0.20; 95% CI: 0.18–0.21), and South-East Asia Region (0.15; 95% CI: 0.14–0.16). Stratification by income level revealed a clear gradient, with the highest ER^ in low-income countries (0.28; 95% CI: 0.27–0.30), followed by lower-middle-income (0.16; 95% CI: 0.15–0.17), upper-middle-income (0.15; 95% CI: 0.14–0.16), and high-income countries (0.08; 95% CI: 0.07–0.08). Corresponding ER* values ranged from 0.04 (95% CI: 0.02–0.07) in high-income to 0.21 (95% CI: 0.14–0.30) in low-income settings.

For psychological IPV, the highest ER^ was reported in the Region of the Americas (0.45; 95% CI: 0.44–0.47) and Eastern Mediterranean Region (0.45; 95% CI: 0.43–0.47), followed by the African Region (0.33; 95% CI: 0.32–0.34). Adjusted ER* estimates ranged from 0.15 (95% CI: 0.08–0.24) in Europe to 0.50 (95% CI: 0.41–0.58) in the Eastern Mediterranean. Across income strata, upper-middle-income countries exhibited the highest prevalence (ER^ = 0.41; 95% CI: 0.40–0.42; ER* = 0.42; 95% CI: 0.33–0.50), while high-income countries reported the lowest (ER^ = 0.14; 95% CI: 0.13–0.15; ER* = 0.11; 95% CI: 0.007–0.18).

For sexual IPV, the African Region had the highest ER^ (0.24; 95% CI: 0.23–0.25), followed by the Eastern Mediterranean (0.18; 95% CI: 0.17–0.19) and South-East Asia (0.16; 95% CI: 0.14–0.16). Corresponding ER* estimates ranged from 0.03 (95% CI: 0.02–0.05) in the Americas to 0.17 (95% CI: 0.13–0.22) in the Eastern Mediterranean. By income level, lower-middle-income countries showed the highest prevalence (ER^ = 0.21; 95% CI: 0.20–0.22; ER* = 0.13; 95% CI: 0.10–0.17), while high-income countries had the lowest (ER^ = 0.08; 95% CI: 0.07–0.08; ER* = 0.05; 95% CI: 0.03–0.09).

For any IPV, the African Region again exhibited the highest ER^ (0.42; 95% CI: 0.41–0.43) and ER* (0.37; 95% CI: 0.30–0.44), followed by the Eastern Mediterranean Region (ER^ = 0.45; 95% CI: 0.44–0.47; ER* = 0.51; 95% CI: 0.42–0.59). The lowest values were observed in the European Region (ER^ = 0.14; 95% CI: 0.13–0.14; ER* = 0.16; 95% CI: 0.10–0.26) and high-income countries (ER^ = 0.13; 95% CI: 0.13–0.13; ER* = 0.17; 95% CI: 0.12–0.23).

In all subgroup analyses, heterogeneity was extremely high (I^2^ > 95%; *p* < 0.001), indicating substantial variability across studies.

### 3.4. Quality Assessment

Overall, the majority of studies received a moderate-to-high quality rating, indicating a generally acceptable level of methodological rigor. Only a small number of studies were classified as low quality (n = 9) (Bikinesi et al., 2017; Gharacheh et al., 2015; Gómez Aristizábal et al., 2022; Gul et al., 2013; Hooker & Taft, 2021; Koirala, 2022; Rishal et al., 2020; Shannon et al., 2016; Wokoma et al., 2014). These lower-rated studies primarily exhibited limitations related to sample representativeness and sample size, or weaknesses in the comparability domain, such as insufficient control for potential confounders. These findings highlight the need for improved study designs and reporting in future research, particularly in ensuring adequate sampling strategies and appropriate adjustment for confounding variables. In the RCTs, the main limitation was potential performance bias due to lack of blinding of participants and personnel. While this methodological shortcoming is often unavoidable in psychological and behavioral interventions—where blinding is inherently difficult to implement—it nevertheless represents a possible source of bias that should be considered when interpreting the results.

## 4. Discussion

### 4.1. Interpretation of the Main Results

This systematic review included 98 studies published between 2013 and 2023, conducted in more than 40 countries worldwide. The most common study design was the CSS, followed by the cohort study. In the studies analyzed, the most commonly used tools to assess intimate partner violence against women during pregnancy and the puerperium were the WHO Women’s Health and Life Experiences Questionnaire (WHO-WHLEQ), Abuse Assessment Screen (AAS), WHO Violence Against Women Instrument (VAWI), and Conflict Tactics Scale (CTS/CTS2). The meta-analysis, conducted using a random-effects model, found the following event rates: 10% (95% CI: 8–12%) for physical violence, 26% (95% CI: 22–31%) for psychological violence, 9% (95% CI: 7–11%) for sexual violence, 16% (95% CI: 5–40%) for verbal violence, and 13% (95% CI: 6–27%) for economic violence. Considering all types of violence together, the event rate for any IPV was 26% (95% CI: 22–30%). Sensitivity analyses stratified by WHO regions showed considerable differences in the prevalence of various types of violence, which were generally higher in the African and Eastern Mediterranean regions, particularly for physical and sexual violence. Psychological violence, on the other hand, was more common in the Region of the Americas and the Eastern Mediterranean Region. The analyses that were carried out according to the World Bank’s income classification showed that high-income countries always had the lowest prevalence of any violence.

Despite the rigorous methodological approach and stratified analyses, the findings of this review must be interpreted with caution due to the consistently high heterogeneity observed across all models (I^2^ > 98%). This reflects substantial variability in study design, population characteristics, IPV definitions, recall periods, and the screening tools adopted. Subgroup analyses by region and income level only partially accounted for this variability. Notably, even within high-income countries, large differences in prevalence persisted, suggesting that cultural, structural, and healthcare system factors may influence IPV disclosure. Therefore, pooled prevalence estimates should be regarded as indicative rather than definitive, underscoring the need for localized, context-sensitive approaches and greater standardization in future research.

### 4.2. Implications for Policies and Practices

The results of this study show that a substantial proportion of women are affected by intimate partner violence during pregnancy and the puerperium. Despite the common perception of pregnancy as a safe and joyful stage of life, it can be a time of significant stress and threat for over one in four women. Many women are unaware that such violence may occur during this period, as highlighted by a recent study on parental information needs and expectations (Brunelli et al., 2023).

The magnitude of this phenomenon is even more concerning when one considers the domino effect that this condition can have not only on the primary object of violence, i.e., the women and the mother, but also on the fetus and the newborn, given how crucial the first 1000 days of life (Draper et al., 2024) are for the future physical and mental health of the baby. Indeed, violence can have particularly detrimental effects during these early years, as the brain and essential functions—including executive function and self-regulation—develop rapidly and in close interaction with the environment (Nelson & Gabard-Durnam, 2020).

For these reasons, the issue should be more effectively addressed in health promotion and prevention programs during pregnancy and the puerperium, as well as in maternal and child healthcare. This requires well-informed and adequately trained healthcare professionals (Kirk & Bezzant, 2020) throughout pregnancy and infancy, including midwives, nurses, health assistants, obstetricians, anesthesiologists, general practitioners, neonatologists, and pediatricians. Asking women this hard question should be taught to all health professionals since academic courses to improve confidence in working with pregnant women who disclose domestic and intimate partner violence (Smith et al., 2018). In addition, the public health system should enable routine screening for all forms of IPV at every stage of the care pathway (e.g., consultations, examinations, follow-up visits) and across all care settings (e.g., hospitals, clinics, primary care, prevention units).

In low- and middle-income countries, the implementation of IPV screening may face unique structural and sociocultural challenges, including limited privacy during consultations, overburdened healthcare systems, insufficient training of staff, and legal frameworks that may not adequately protect women. These barriers can significantly affect the feasibility of routine screening and the willingness of women to disclose violence, highlighting the need for locally adapted and context-sensitive approaches.

In this sense, the use of validated violence detection tools that are culturally adapted as needed, such as the WHO-WHLEQ, AAS, and VAWI, is essential. However, IPV screening must be conducted with full respect for women’s confidentiality and safety, ensuring that disclosures occur freely and without influence from the potential perpetrator. Opportunities to be explored include access to health services, e.g., blood tests, stress curves for diabetes screening, pap smear and swab collection, vaccinations, and antenatal classes, when the woman is alone more often. Alternatively, private and confidential spaces should be created to facilitate disclosure during other healthcare encounters. Furthermore, public awareness campaigns are needed to reduce stigma and encourage help-seeking among women experiencing IPV during pregnancy and the puerperium.

In any case, close collaboration with other health professionals, social services, anti-violence centers and services, and law enforcement agencies, as well as the establishment of multi-professional intervention protocols, is essential to ensure a rapid and efficient response to any form of violence.

Moreover, IPV during pregnancy and the puerperium should be fully recognized as a public health priority and integrated into national and international guidelines for early identification and response. Furthermore, systematic monitoring and evaluation of interventions to detect and address IPV during this period should be implemented through a dedicated surveillance system.

### 4.3. Future Directions

This analysis highlights the need for further research to address several outstanding questions and areas for improvement. First, a comparative analysis of existing screening tools should be conducted to determine the most accurate—considering sensitivity, specificity, and positive and negative predictive values—but also acceptable tool for women and health professionals. Secondly, the effectiveness of alternative digital screening tools, such as the use of apps or online questionnaires integrated into telemedicine pathways or remote prenatal care, needs to be further investigated. Thirdly, intervention studies are needed to evaluate the real-world impact of implementing systematic IPV screening during pregnancy and the puerperium, particularly with regard to standard care and maternal and child health outcomes. Finally, the cost-effectiveness of introducing such screening should be assessed from a public health perspective to better inform health policy.

### 4.4. Strengths and Limitations

This systematic review and meta-analysis have several strengths. First, it is the most comprehensive synthesis to date focusing specifically on validated screening tools for the detection of IPV during pregnancy and the puerperium. The inclusion of 98 studies from over 40 countries ensures broad geographic and cultural representation and increases the generalizability of the results. The rigorous methodology, adherence to PRISMA guidelines, and registration of the protocol in PROSPERO contribute to the transparency and reproducibility of the review. Moreover, the use of subgroup meta-analyses by type of violence (physical, psychological, sexual, verbal, economic, and non-physical) allows for a nuanced understanding of the prevalence of different IPV forms across various settings. The assessment of publication bias and heterogeneity using appropriate statistical methods further strengthens the reliability of the results. While this review cataloged the screening tools used, a critical appraisal of their psychometric properties was not performed, as it was beyond the scope defined in our protocol, and such data are typically reported in validation studies rather than in prevalence-focused research.

However, some limitations should be acknowledged. High heterogeneity (I^2^ > 90%) was observed in most meta-analyses, likely due to variability in study designs, populations, settings, and screening tools. Although only validated instruments were included, differences in item wording, recall periods, and cultural adaptations may have affected comparability. Most of the included studies were cross-sectional, which limits the ability to infer temporal or causal relationships. One limitation worth mentioning is the potential bias in performance due to the lack of blinding of participants and staff in the included trials. While this methodological shortcoming is often unavoidable in psychological and behavioral interventions—as blinding is inherently difficult to implement—it nevertheless represents a potential source of bias that should be considered when interpreting the results. Although studies were grouped by IPV subtype, the presence of co-occurring IPV forms cannot be excluded. Most studies assessed multiple IPV types, but not all provided data on their overlap. Therefore, prevalence estimates by subtype should be interpreted with caution. Moreover, only articles published in English or Italian were considered, which may introduce linguistic bias. Lastly, publication bias was identified in several outcome categories, which may have led to an over- or underestimation of prevalence rates. Importantly, the consistent presence of high heterogeneity and the limited availability of detailed psychometric data also imply that recommendations on the systematic implementation of IPV screening should be interpreted with caution. While the goal of integrating routine screening into care pathways is essential, real-world application faces ethical, logistical, and contextual barriers, especially in low-resource settings, that require further investigation and system-level preparedness.

While our review focused on empirical applications of IPV screening tools, it is worth noting that some instruments were informed by theoretical constructs, such as the ecological model or stages of change, though this information was not consistently reported across studies. Future research could benefit from a more explicit linkage between theory and tool development or application.

## 5. Conclusions

This systematic review and meta-analysis highlight the high prevalence of IPV during pregnancy and the puerperium, with psychological, physical, and sexual forms being the most commonly reported. Despite the differences between countries and settings, the findings underline the global relevance of the issue and the urgent need for routine screening in maternal healthcare. Validated tools—such as the WHO-WHLEQ, AAS, and VAWI—have been shown to be widely used and adaptable in different contexts, supporting their implementation in clinical and community settings. However, the heterogeneity of study design and tool characteristics calls for greater standardization in future research. The integration of effective screening tools into antenatal and postnatal care can play a critical role in early detection, timely support, and improved health outcomes for women and their children.

## Figures and Tables

**Table 1:** Eligibility criteria used to identify relevant studies, according to the PECOS.

Search Strategy	Details
Inclusion criteria	P: Pregnant women (across all trimesters) or women in the puerperium period. E: Any type of domestic violence perpetrated during pregnancy by an intimate partner. C: Not applicable. O: Prevalence of violence; type of screening tool. S: Original observational studies (including cross-sectional, case–control, or cohort, both prospective and retrospective studies) or interventional studies published as peer-reviewed articles in international scientific journals.
Exclusion criteria	P: Nulliparas, menopausal women, pre-pubertal females. E: Other types of violence not perpetrated during pregnancy and not by an intimate partner. C: Not applicable. O: Any other health outcomes. S: Not original (reviews with or without meta-analysis), not performed among humans, not published as peer-reviewed articles in international scientific journals (book, book chapter, thesis), no full-text papers (abstract, conference paper, letter, commentary, note).
Language	English and Italian
Time filter	Last 10 years

## Data Availability

We will share the research data with MDPI.

## Supplementary Materials

The following supporting information can be downloaded at https://www.mdpi.com/article/10.3390/ejihpe15080161/s1: Figure S1: (a) A forest plot and (b) funnel plot of the random-effects model assessing physical IPV; Figure S2: (a) A forest plot and (b) funnel plot of the random-effects model assessing psychological IPV; Figure S3: (a) A forest plot and (b) funnel plot of the random-effects model assessing sexual IPV; Figure S4: (a) A forest plot and (b) funnel plot of the random-effects model assessing any IPV. Table S1: Full search strategy for each database; Table S2: Summary statistics of random-effects and fixed-effects models; Table S3: Summary statistics of random-effects and fixed-effects models: Sensitivity analyses by WHO region; Table S4: Summary statistics of random-effects and fixed-effects models: Sensitivity analyses by country income level.
