# Supplementary material for: Prevalence and Screening Tools of Intimate Partner Violence Among Pregnant and Postpartum Women: A Systematic Review and Meta-Analysis
Source: Eur J Investig Health Psychol Educ. 2025 Aug 15;15(8):161. doi: 10.3390/ejihpe15080161 (PMC12385551; doi:10.3390/ejihpe15080161)
Supplement: Supplementary file 1 [file ejihpe-15-00161-s001.zip › ejihpe-3703202-supplementary-edited.pdf]

Supplementary table 1. Full search strategy for each database.

Search strategy	Database	N. records
("Intimate Partner Violence"[MeSH Terms] OR "Violence"[MeSH Terms] OR "Domestic Violence"[MeSH Terms] OR "Physical Abuse"[MeSH Terms] OR "Gender-Based Violence"[MeSH Terms] OR "Intimate Partner Violence"[Title/Abstract] OR "Violence"[Title/Abstract] OR "Domestic Violence"[Title/Abstract] OR "Physical Abuse"[Title/Abstract] OR "Gender-Based Violence"[Title/Abstract]) AND ("pregnancy"[MeSH Terms] OR "postpartum period"[MeSH Terms] OR "pregnan*"[Title/Abstract] OR "gestation*"[Title/Abstract] OR "puerper*"[Title/Abstract])	PubMed/Medline	8,350
('physical abuse'/exp OR 'physical abuse' OR 'violence'/exp OR 'violence' OR 'gender based violence'/exp OR 'gender based violence' OR 'domestic violence'/exp OR 'domestic violence' OR 'partner violence'/exp OR 'partner violence') AND (gestation OR 'post AND partum AND period' OR 'puerperium'/exp OR 'puerperium' OR 'pregnancy'/exp OR 'pregnancy')	EMBASE	11,993
((TITLE-ABS-KEY (pregnan*) OR TITLE-ABS-KEY (gestation*) OR TITLE-ABS-KEY (puerper*))) AND (((TITLE-ABS-KEY (violence) OR TITLE-ABS-KEY (abuse))) AND ((TITLE-ABS-KEY (intimate AND partner) OR TITLE-ABS-KEY (gender-based) OR TITLE-ABS-KEY (domestic) OR TITLE-ABS-KEY (physical))))	Scopus	7,114
(TS=(Gender-Based Violence) OR TS=(Physical Abuse) OR TS=(Domestic Violence) OR TS=(Intimate Partner Violence) OR TS=(violence)) AND (TS=(gestation*) OR TS=(post-partum) OR TS=(post partum) OR TS=(pot partum) OR TS=(puerper*) OR TS=(pregnancy) OR TS=(pregnan*))	Web of Science	7,263

Fig. S1. (a) A forest plot and (**b)** funnel plot of the random-effects model assessing physical IPV.

a)

	ES	95% CI	Sig.	N
Abdollahi 2015	0.14	0.12 , 0.16	0.000	1461
Abebe 2016	0.29	0.24 , 0.35	0.000	282
Abujilban 2022	0.31	0.26 , 0.37	0.000	247
Almeida 2017	0.22	0.19 , 0.25	0.000	852
Antoniou 2019	0.02	0.01 , 0.04	0.000	546
Aristizabal 2022	0.12	0.10 , 0.14	0.000	1447
Asiimwe 2022	0.32	0.24 , 0.42	0.000	100
Atilla 2023	0.07	0.05 , 0.10	0.000	456
Avci 2023	0.15	0.11 , 0.20	0.000	255
Bagcioglu 2014	0.05	0.03 , 0.08	0.000	317
Bahrami-Vazir 2020	0.22	0.19 , 0.26	0.000	525
Belay 2019	0.09	0.07 , 0.12	0.000	589
Bernstein 2016	0.15	0.12 , 0.18	0.000	623
Bikinesi 2017	0.03	0.02 , 0.05	0.000	386
Boonnate 2015	0.03	0.01 , 0.06	0.000	230
De Lima 2016	0.03	0.02 , 0.05	0.000	359
Dinmohammadi 2021	0.18	0.09 , 0.33	0.000	41
Elkhateeb 2021	0.30	0.26 , 0.34	0.000	513
Farrokh-Eslamlou 2014	0.10	0.07 , 0.14	0.000	313
Fekadu 2018	0.32	0.28 , 0.36	0.000	450
Ferdos 2017	0.39	0.35 , 0.44	0.000	443
Field 2018	0.76	0.71 , 0.80	0.000	376
Fonseca-Machado 2015	0.36	0.31 , 0.41	0.000	358
Gebrekristos 2023	0.17	0.11 , 0.26	0.000	90
Gharacheh 2016	0.26	0.22 , 0.31	0.000	328
Gul 2013	0.36	0.28 , 0.45	0.002	129
Ibri 2023	0.39	0.33 , 0.45	0.001	240
Islam 2018	0.35	0.31 , 0.40	0.000	426
Kana 2020	0.34	0.29 , 0.40	0.000	293
Khalroniuk 2017	0.13	0.11 , 0.15	0.000	1200
Khatlani 2023	0.10	0.08 , 0.12	0.000	795
Kita 2016	0.02	0.01 , 0.04	0.000	562
Kita 2017	0.03	0.02 , 0.05	0.000	453
Koirala 2022	0.29	0.23 , 0.35	0.000	220
Krishnamurti (a) 2021	0.00	0.00 , 0.01	0.000	552
Krishnamurti (b) 2021	0.01	0.00 , 0.02	0.000	407
Lee 2023	0.00	0.00 , 0.00	0.000	5616
Lima 2020	0.18	0.10 , 0.29	0.000	65
Luhumyo 2020	0.23	0.19 , 0.28	0.000	369
Lukasse 2014	0.02	0.02 , 0.02	0.000	7174
Mahenge 2013	0.18	0.16 , 0.20	0.000	1180
Martin-de-las-Heras 2015	0.04	0.03 , 0.06	0.000	779
Martin-de-las-Heras 2019	0.04	0.03 , 0.06	0.000	779
McKelvie 2021	0.11	0.07 , 0.16	0.000	188
Mohamed 2013	0.28	0.24 , 0.33	0.000	404
Musa 2020	0.26	0.23 , 0.30	0.000	648
Muzrif 2018	0.06	0.05 , 0.07	0.000	2088
Naghizadeh 2021	0.05	0.03 , 0.09	0.000	250
Nhi 2019	0.03	0.02 , 0.04	0.000	1274
Njoku 2021	0.45	0.40 , 0.50	0.046	400
Okunola 2021	0.04	0.02 , 0.07	0.000	363
Omoronyia 2020	0.27	0.22 , 0.33	0.000	250
Priya 2019	0.60	0.52 , 0.67	0.011	165
Pun 2018	0.04	0.03 , 0.05	0.000	1011
Pun 2019	0.02	0.01 , 0.03	0.000	1381
Rasch (a) 2018	0.06	0.05 , 0.08	0.000	1116
Rasch (b) 2018	0.03	0.02 , 0.04	0.000	1309
Rees 2016	0.06	0.05 , 0.07	0.000	1672
Ribeiro 2017	0.12	0.10 , 0.14	0.000	1446
Ribeiro 2019	0.12	0.10 , 0.14	0.000	1139
Samal 2022	0.05	0.03 , 0.09	0.000	200
Sanchez 2023	0.02	0.01 , 0.03	0.000	600
Sapkota 2021	0.12	0.08 , 0.19	0.000	140
Shamu 2013	0.06	0.05 , 0.07	0.000	1951
Shamu 2014	0.06	0.05 , 0.07	0.000	1951
Shannon 2015	0.32	0.23 , 0.43	0.002	77
Shrestha 2016	0.03	0.02 , 0.05	0.000	404
Silva 2020	0.08	0.06 , 0.11	0.000	330
Silva 2022	0.07	0.05 , 0.10	0.000	327
Sobhani 2018	0.10	0.07 , 0.13	0.000	402
Sulaiman 2021	0.22	0.18 , 0.26	0.000	403
Taiwiie 2023	0.04	0.03 , 0.06	0.000	473
Utaile 2023	0.34	0.32 , 0.36	0.000	1635
Velasco (a) 2014	0.02	0.01 , 0.03	0.000	779
Velasco (b) 2014	0.04	0.03 , 0.06	0.000	779
Wangel 2016	0.14	0.12 , 0.16	0.000	1003
Watson 2013	0.10	0.09 , 0.12	0.000	1726
Wokoma (a) 2014	0.05	0.03 , 0.07	0.000	507
Wokoma (b) 2014	0.01	0.00 , 0.02	0.000	507
Zapata-Calvente 2022	0.05	0.04 , 0.07	0.000	592
Zheng 2020	0.01	0.01 , 0.02	0.000	813
Overall (random-effects model)	0.10	0.08 , 0.12	0.000	63429

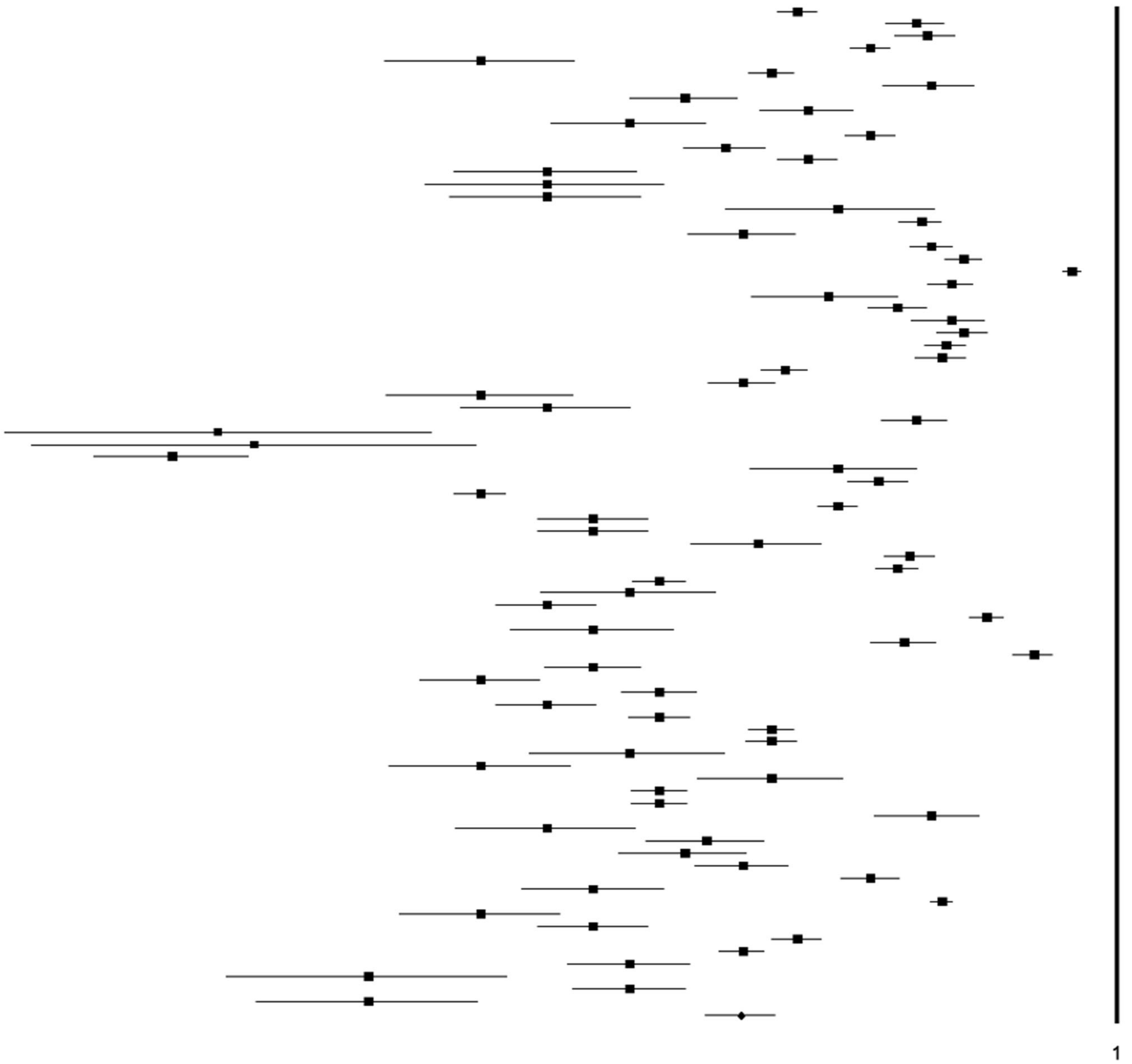

b)

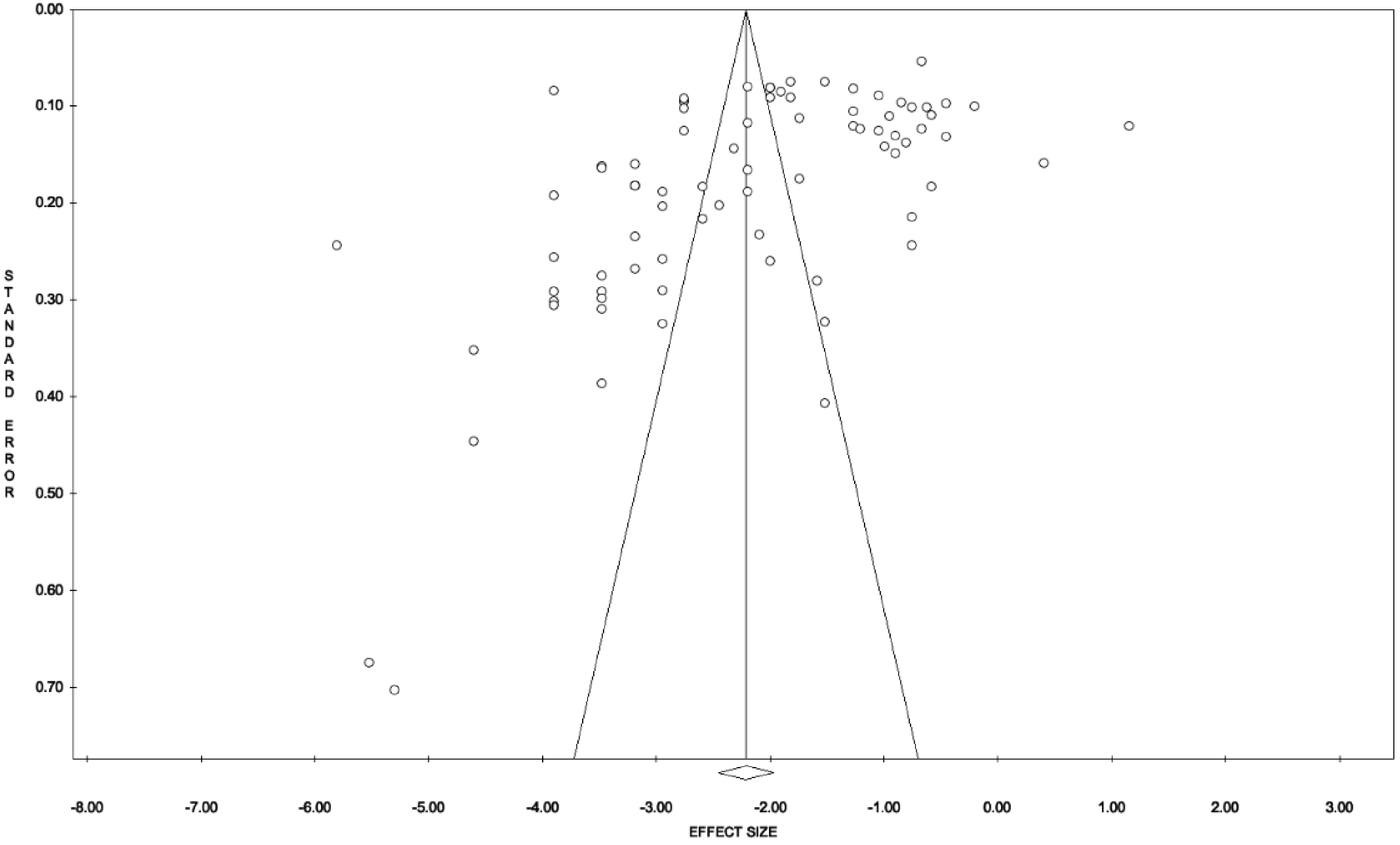

Interpretive note:

The forest plot (Figure S1a) illustrates the estimated prevalence of physical intimate partner violence (IPV) across included studies, with most estimates showing consistency in direction and magnitude. The size of each square reflects the weight of the corresponding study, and the pooled estimate (diamond) supports a significant overall effect (event rate of 0.10, 95% CI: 0.08–0.12, $p < 0.001$). The funnel plot (Figure S1b) shows slight asymmetry, suggesting potential publication bias or small-study effects.

Fig. S2. (a) A forest plot and (**b)** funnel plot of the random-effect models assessing psychological IPV.

a)

	ES	95% CI	Sig.	N
Abebe 2016	0.16	0.12 , 0.21	0.000	282
Abujilban 2022	0.66	0.60 , 0.72	0.000	247
Almeida 2017	0.43	0.40 , 0.46	0.000	852
Antoniou 2019	0.03	0.02 , 0.05	0.000	546
Aristizabal 2022	0.48	0.45 , 0.51	0.128	1447
Atilla 2023	0.33	0.29 , 0.37	0.000	456
Avci 2023	0.39	0.33 , 0.45	0.000	255
Bahrami-Vazir 2020	0.39	0.35 , 0.43	0.000	525
Belay 2019	0.15	0.12 , 0.18	0.000	589
Bernstein 2016	0.15	0.12 , 0.18	0.000	623
Bikinesi 2017	0.07	0.05 , 0.10	0.000	386
Debono 2017	0.15	0.11 , 0.20	0.000	300
Dinmohammadi 2021	0.56	0.45 , 0.66	0.278	82
Elkhateeb 2021	0.45	0.41 , 0.49	0.024	513
Farokh-Eslamlou 2014	0.43	0.38 , 0.49	0.014	313
Fekadu 2018	0.58	0.53 , 0.62	0.001	450
Field 2018	0.81	0.77 , 0.85	0.000	376
Fonseca-Machado 2015	0.95	0.92 , 0.97	0.000	358
Gebrekristos 2023	0.37	0.28 , 0.47	0.015	90
Gharacheh 2016	0.88	0.84 , 0.91	0.000	328
Gul 2013	0.46	0.38 , 0.55	0.364	129
Ilori 2023	0.40	0.34 , 0.46	0.002	240
Islam 2018	0.65	0.60 , 0.69	0.000	426
Iyengar 2020	0.43	0.34 , 0.52	0.126	120
Kana 2020	0.51	0.45 , 0.57	0.732	293
Khaironisak 2017	0.30	0.27 , 0.33	0.000	1200
Khatlani 2023	0.39	0.36 , 0.42	0.000	795
Koirala 2022	0.31	0.25 , 0.37	0.000	220
Krishnamurti (a) 2021	0.01	0.00 , 0.02	0.000	552
Krishnamurti (b) 2021	0.01	0.00 , 0.03	0.000	407
Lee 2023	0.03	0.03 , 0.03	0.000	5616
Lima 2020	0.40	0.29 , 0.52	0.109	65
Luhumyo 2020	0.27	0.23 , 0.32	0.000	369
Lukasse 2014	0.03	0.03 , 0.03	0.000	7174
Martin-de-las-Heras 2015	0.21	0.18 , 0.24	0.000	779
Martin-de-las-Heras 2019	0.21	0.18 , 0.24	0.000	779
McKelvie 2021	0.39	0.32 , 0.46	0.003	188
Mohamed 2013	0.39	0.34 , 0.44	0.000	404
Musa 2020	0.26	0.23 , 0.30	0.000	648
Naghizadeh 2021	0.33	0.27 , 0.39	0.000	250
Nhi 2019	0.32	0.29 , 0.35	0.000	1274
Njoku 2021	0.34	0.30 , 0.39	0.000	400
Okunola 2021	0.04	0.02 , 0.07	0.000	363
Omoronyia 2020	0.51	0.45 , 0.57	0.752	250
Pun 2019	0.05	0.04 , 0.06	0.000	1381
Rasch (a) 2018	0.23	0.21 , 0.26	0.000	1116
Rasch (b) 2018	0.32	0.30 , 0.35	0.000	1309
Rees 2016	0.31	0.29 , 0.33	0.000	1672
Rees 2017	0.31	0.29 , 0.33	0.000	1672
Ribeiro 2017	0.48	0.45 , 0.51	0.128	1446
Ribeiro 2019	0.47	0.44 , 0.50	0.043	1139
Samal 2022	0.01	0.00 , 0.04	0.000	200
Shamu 2013	0.44	0.42 , 0.46	0.000	2042
Shamu 2014	0.18	0.16 , 0.20	0.000	1951
Shannon 2015	0.71	0.60 , 0.80	0.000	77
Shrestha 2016	0.17	0.14 , 0.21	0.000	404
Silva 2020	0.16	0.12 , 0.20	0.000	330
Silva 2022	0.17	0.13 , 0.21	0.000	327
Sobhani 2018	0.45	0.40 , 0.50	0.045	402
Takelle 2023	0.06	0.04 , 0.09	0.000	473
Utaile 2023	0.35	0.33 , 0.37	0.000	1535
Velasco (a) 2014	0.05	0.04 , 0.07	0.000	779
Velasco (b) 2014	0.21	0.18 , 0.24	0.000	779
Watson 2013	0.13	0.11 , 0.15	0.000	1726
Wokoma (a) 2014	0.10	0.08 , 0.13	0.000	507
Wokoma (b) 2014	0.02	0.01 , 0.04	0.000	507
Zapata-Calvente 2022	0.19	0.16 , 0.22	0.000	592
Zheng 2020	0.11	0.09 , 0.13	0.000	813
Zou 2015	0.64	0.57 , 0.70	0.000	223
Overall (random-effects model)	0.26	0.22 , 0.31	0.000	55361

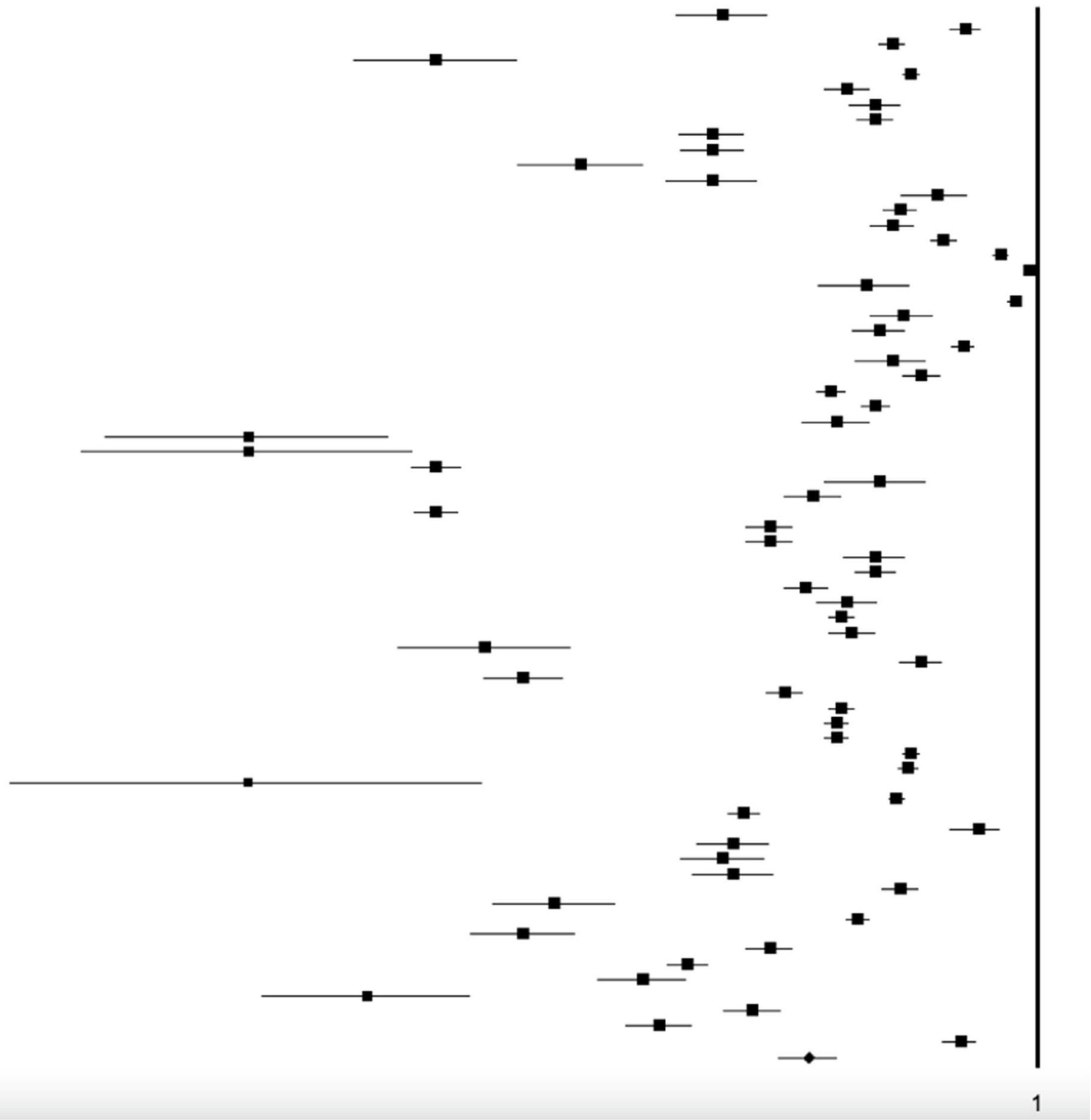

b)

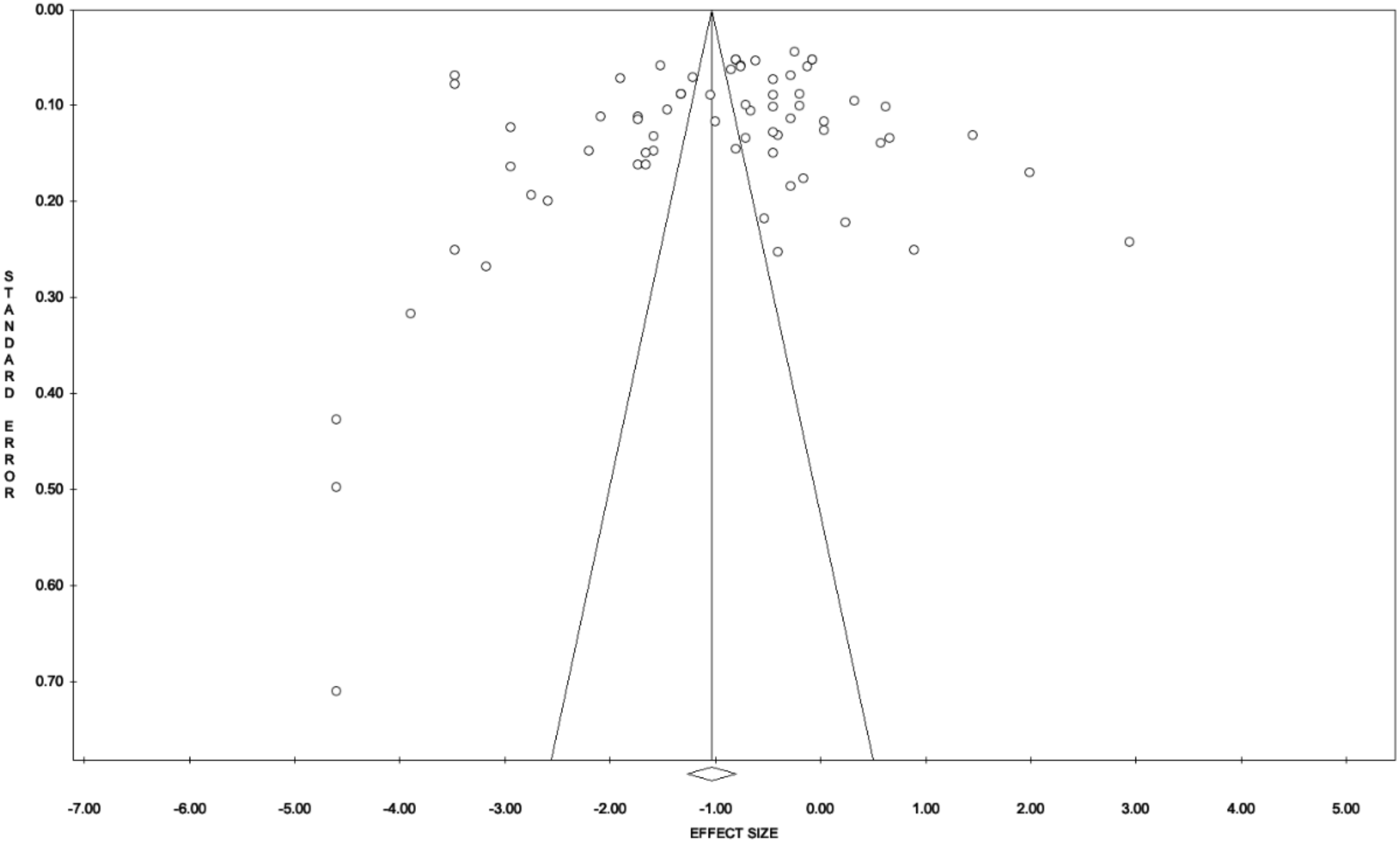

Interpretive note:

The forest plot (Figure S2a) shows generally consistent prevalence estimates of psychological intimate partner violence (IPV) across studies, with the pooled effect (diamond) indicating a significant overall prevalence (event rate of 0.26, 95% CI: 0.22–0.31, $p < 0.001$). The funnel plot (Figure S2b) displays slight asymmetry, suggesting possible publication bias or small-study effects. These visualizations support the robustness of the findings while highlighting some heterogeneity across studies.

Fig. S3. (a) A forest plot and (**b)** funnel plot of the random-effects model assessing sexual IPV.

a)

	ES	95% CI	Sig.	N
Abebe 2016	0.30	0.25 , 0.36	0.000	282
Abujilban 2022	0.09	0.06 , 0.13	0.000	247
Almeida 2017	0.20	0.17 , 0.23	0.000	852
Antoniou 2019	0.02	0.01 , 0.04	0.000	546
Asiimwe 2022	0.45	0.36 , 0.55	0.318	100
Atilla 2023	0.06	0.04 , 0.09	0.000	456
Avci 2023	0.07	0.04 , 0.11	0.000	255
Bagcioglu 2014	0.05	0.03 , 0.08	0.000	317
Bahrami-Vazir 2020	0.20	0.17 , 0.24	0.000	525
Belay 2019	0.09	0.07 , 0.12	0.000	589
Bernstein 2016	0.02	0.01 , 0.03	0.000	623
Bikinesi 2017	0.02	0.01 , 0.04	0.000	386
De Lima 2016	0.01	0.00 , 0.03	0.000	359
Dinmohammadi 2021	0.27	0.19 , 0.38	0.000	82
Elkhateeb 2021	0.20	0.17 , 0.24	0.000	513
Farrokh-Eslamlou 2014	0.17	0.13 , 0.22	0.000	313
Fekadu 2018	0.08	0.06 , 0.11	0.000	450
Ferdos 2017	0.26	0.22 , 0.30	0.000	443
Field 2018	0.26	0.22 , 0.31	0.000	376
Fonseca-Machado 2015	0.02	0.01 , 0.04	0.000	358
Gharacheh 2016	0.35	0.30 , 0.40	0.000	328
Gul 2013	0.20	0.14 , 0.28	0.000	129
Ilori 2023	0.38	0.32 , 0.44	0.000	240
Islam 2018	0.18	0.15 , 0.22	0.000	426
Kana 2020	0.31	0.26 , 0.37	0.000	293
Khaironisak 2017	0.10	0.08 , 0.12	0.000	1200
Khatlani 2023	0.10	0.08 , 0.12	0.000	795
Koirala 2022	0.23	0.18 , 0.29	0.000	220
Krishnamurti (a) 2021	0.04	0.03 , 0.06	0.000	552
Krishnamurti (b) 2021	0.02	0.01 , 0.04	0.000	407
Lima 2020	0.03	0.01 , 0.11	0.000	65
Luhumyo 2020	0.13	0.10 , 0.17	0.000	369
Lukasse 2014	0.04	0.04 , 0.04	0.000	7174
Mahenge 2013	0.20	0.18 , 0.22	0.000	1180
McKelvie 2021	0.07	0.04 , 0.12	0.000	188
Mohamed 2013	0.14	0.11 , 0.18	0.000	404
Musa 2020	0.04	0.03 , 0.06	0.000	648
Naghizadeh 2021	0.12	0.09 , 0.17	0.000	250
Nhi 2019	0.10	0.08 , 0.12	0.000	1274
Njoku 2021	0.07	0.05 , 0.10	0.000	400
Okunola 2021	0.04	0.02 , 0.07	0.000	363
Omoronyia 2020	0.29	0.24 , 0.35	0.000	250
Pun 2019	0.01	0.01 , 0.02	0.000	1381
Pun 2018	0.02	0.01 , 0.03	0.000	1011
Rasch (a) 2018	0.15	0.13 , 0.17	0.000	1116
Rasch (b) 2018	0.10	0.08 , 0.12	0.000	1309
Ribeiro 2017	0.03	0.02 , 0.04	0.000	1446
Samal 2022	0.00	0.00 , 0.04	0.000	200
Sapkota 2021	0.15	0.10 , 0.22	0.000	140
Shamu 2013	0.39	0.37 , 0.41	0.000	2042
Shamu 2014	0.23	0.21 , 0.25	0.000	1951
Shannon 2015	0.14	0.08 , 0.24	0.000	77
Shrestha 2016	0.17	0.14 , 0.21	0.000	404
Silva 2020	0.03	0.02 , 0.06	0.000	330
Silva 2022	0.03	0.02 , 0.06	0.000	327
Sobhani 2018	0.17	0.14 , 0.21	0.000	402
Takelle 2023	0.03	0.02 , 0.05	0.000	473
Utaile 2023	0.19	0.17 , 0.21	0.000	1535
Velasco 2014	0.01	0.00 , 0.02	0.000	779
Wangel 2016	0.15	0.13 , 0.17	0.000	1003
Watson 2013	0.05	0.04 , 0.06	0.000	1726
Zapata-Calvente 2022	0.02	0.01 , 0.04	0.000	592
Zheng 2020	0.01	0.01 , 0.02	0.000	813
Overall (random-effects model)	0.09	0.07 , 0.11	0.000	44284

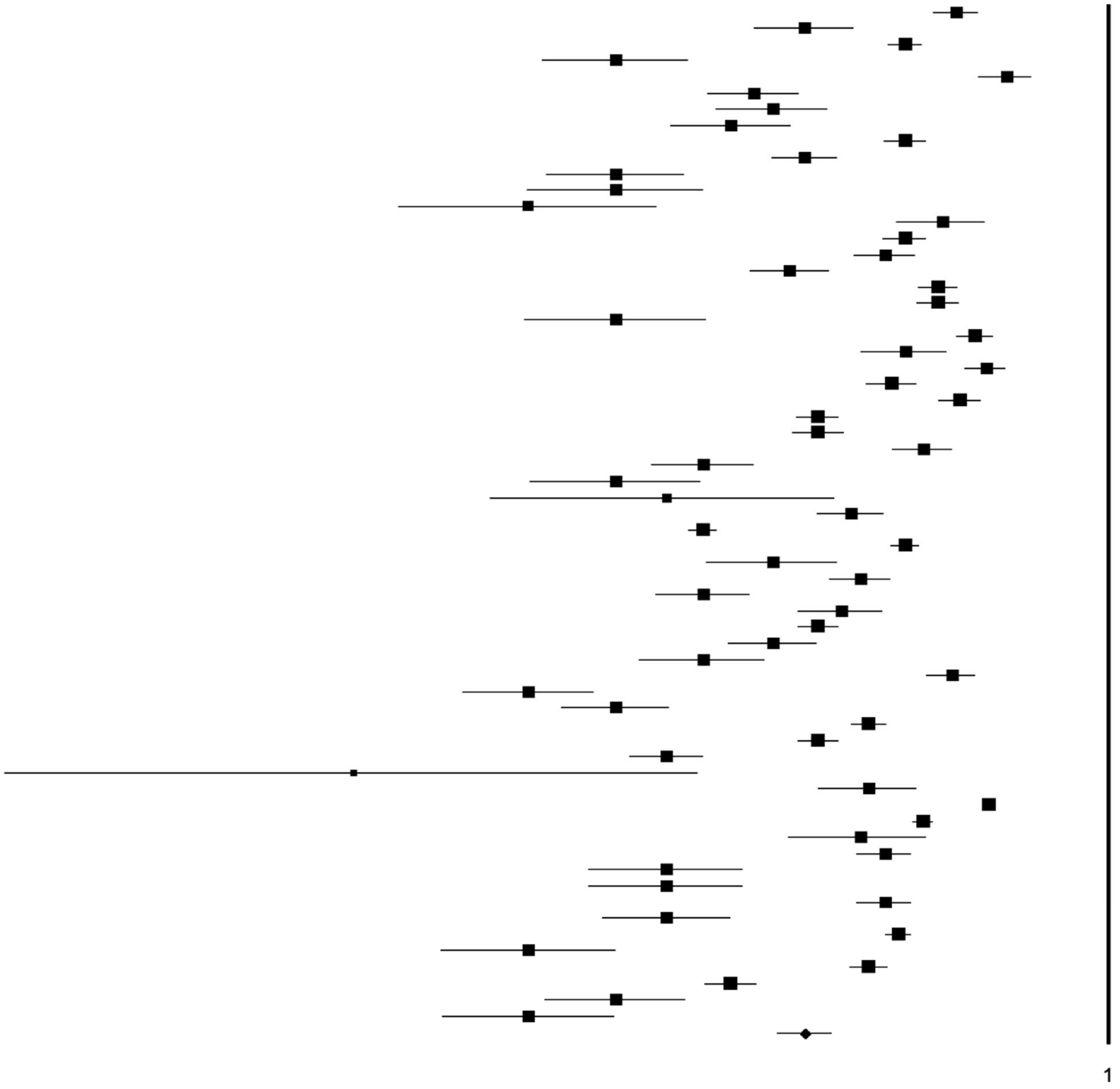

b)

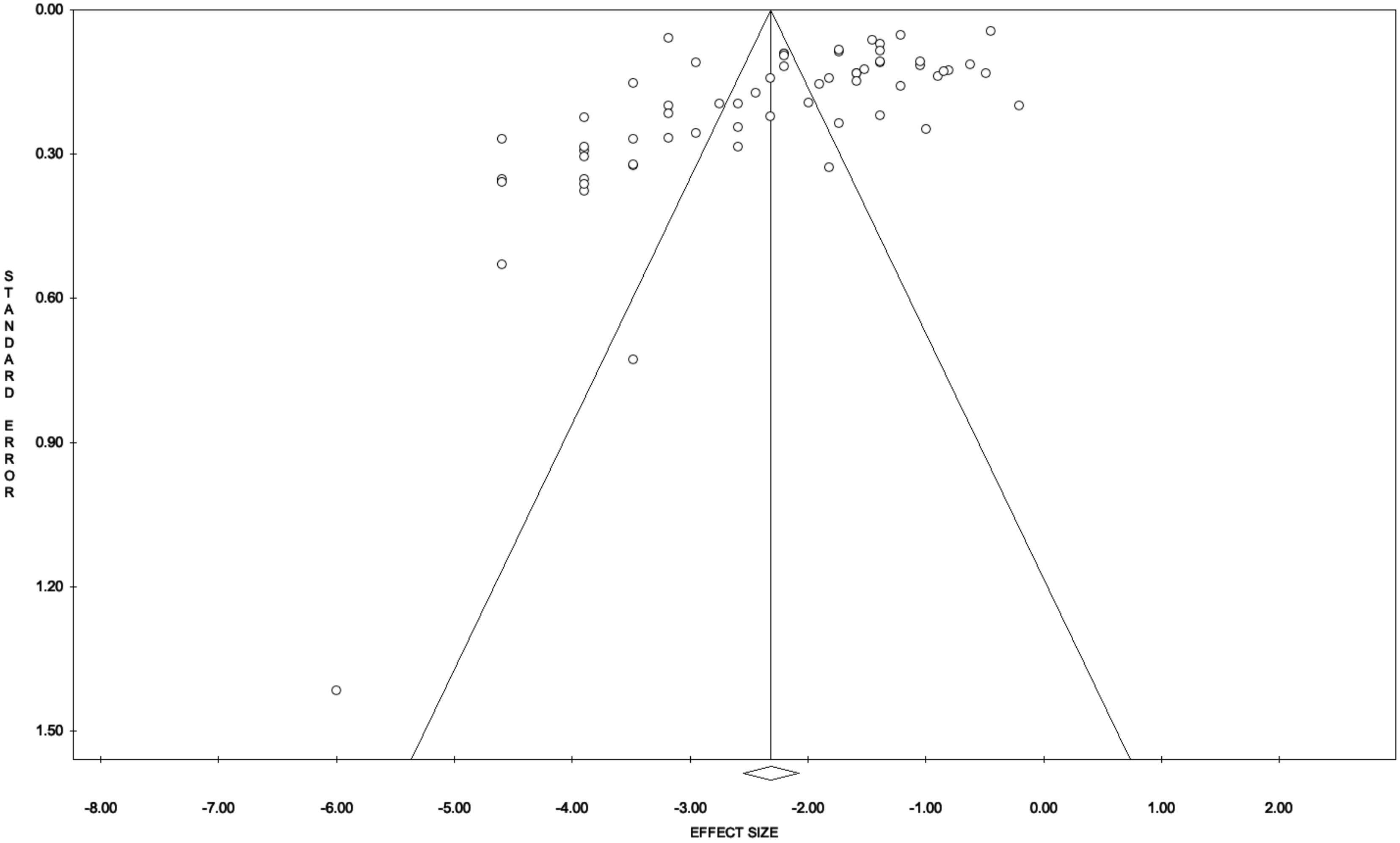

Interpretive note:

The forest plot (Figure S3a) shows generally consistent prevalence estimates of sexual intimate partner violence (IPV) across studies, with the pooled effect (diamond) indicating a significant overall prevalence (event rate of 0.09, 95% CI: 0.07–0.11, $p < 0.001$). The funnel plot (Figure S3b) displays slight asymmetry, suggesting possible publication bias or small-study effects. These visualizations support the robustness of the findings while highlighting some heterogeneity across studies.

Fig. S4. (a) A forest plot and (**b)** funnel plot of the random-effects model assessing any IPV.

a)

	ES	95% CI	Sig.	N
Abebe 2016	0.44	0.38 , 0.50	0.044	282
Abujilban 2022	0.93	0.89 , 0.96	0.000	247
Alhalal 2022	0.29	0.26 , 0.33	0.000	684
Alhusen 2015	0.19	0.14 , 0.26	0.000	166
Almeida 2017	0.43	0.40 , 0.46	0.000	852
Andreasen 2023	0.10	0.10 , 0.10	0.000	16068
Antoniou 2019	0.06	0.04 , 0.08	0.000	546
Aristizabal 2022	0.50	0.47 , 0.53	1.000	1447
Atilla 2023	0.44	0.40 , 0.49	0.011	456
Avci 2023	0.10	0.07 , 0.14	0.000	255
Bagcioglu 2014	0.10	0.07 , 0.14	0.000	317
Belay 2019	0.21	0.18 , 0.24	0.000	589
Bernstein 2016	0.21	0.18 , 0.24	0.000	623
Bikinesi 2017	0.08	0.06 , 0.11	0.000	386
Boonnate 2015	0.12	0.08 , 0.17	0.000	230
Caprara 2020	0.15	0.11 , 0.20	0.000	232
Chen 2017	0.07	0.06 , 0.08	0.000	1438
Dinmohammadi 2021	0.59	0.48 , 0.69	0.105	82
Elkhateeb 2021	0.51	0.47 , 0.55	0.651	513
Farrokh-Eslamlou 2014	0.56	0.50 , 0.61	0.034	313
Fekadu 2018	0.59	0.54 , 0.63	0.000	450
Field 2018	0.15	0.12 , 0.19	0.000	376
Fisher (a) 2013	0.04	0.02 , 0.06	0.000	417
Fisher (b) 2013	0.06	0.04 , 0.09	0.000	453
Fonseca-Machado 2015	0.18	0.14 , 0.22	0.000	358
Gashaw 2019	0.44	0.40 , 0.48	0.001	720
Gebrekristos 2023	0.40	0.30 , 0.50	0.060	90
Gelaye 2016	0.37	0.35 , 0.39	0.000	2970
Gharacheh 2016	0.44	0.39 , 0.49	0.030	328
Hooker 2021	0.07	0.06 , 0.08	0.000	2621
Ilori 2023	0.46	0.40 , 0.52	0.216	240
Iyengar 2020	0.35	0.27 , 0.44	0.001	120
Kana 2020	0.67	0.61 , 0.72	0.000	293
Kataoka 2018	0.19	0.10 , 0.33	0.000	43
Khaironisak 2017	0.36	0.33 , 0.39	0.000	1200
Kian (a) 2019	0.41	0.34 , 0.48	0.011	200
Kian (b) 2019	0.37	0.31 , 0.44	0.000	200
Kita 2017	0.12	0.09 , 0.15	0.000	453
Koirala 2022	0.33	0.27 , 0.39	0.000	220
Lee 2023	0.07	0.06 , 0.08	0.000	5616
Luhumyo 2020	0.34	0.29 , 0.39	0.000	369
Mahenge 2013	0.27	0.25 , 0.30	0.000	1180
Martin-de-las-Heras 2015	0.21	0.18 , 0.24	0.000	779
McKelvie 2021	0.45	0.38 , 0.52	0.171	188
Mohamed 2013	0.52	0.47 , 0.57	0.422	404
Musa 2020	0.40	0.36 , 0.44	0.000	648
Naghizadeh 2021	0.35	0.29 , 0.41	0.000	250
Nhi 2019	0.35	0.32 , 0.38	0.000	1274
Njoku 2021	0.43	0.38 , 0.48	0.005	400
Nongrum 2014	0.07	0.04 , 0.12	0.000	150
Okunola 2021	0.15	0.12 , 0.19	0.000	363
Omoronyia 2020	0.55	0.49 , 0.61	0.114	250
Priya 2019	0.23	0.17 , 0.30	0.000	165
Pun 2018	0.24	0.21 , 0.27	0.000	1011
Pun 2019	0.20	0.18 , 0.22	0.000	1381
Rasch (a) 2018	0.30	0.27 , 0.33	0.000	1116
Rasch (b) 2018	0.35	0.32 , 0.38	0.000	1309
Ribeiro 2017	0.50	0.47 , 0.53	1.000	1446
Rishal 2020	0.18	0.16 , 0.20	0.000	1010
Samal 2022	0.06	0.03 , 0.10	0.000	200
Sanchez (a) 2023	0.07	0.05 , 0.09	0.000	600
Sanchez (b) 2023	0.03	0.02 , 0.05	0.000	600
Shamu 2013	0.63	0.61 , 0.65	0.000	2042
Shamu 2014	0.33	0.31 , 0.35	0.000	1951
Shannon 2015	0.75	0.64 , 0.83	0.000	77
Shrestha 2016	0.27	0.23 , 0.32	0.000	404
Sobhani 2018	0.48	0.43 , 0.53	0.423	402
Sulaiman 2021	0.56	0.51 , 0.61	0.016	403
Sussmann 2020	0.25	0.22 , 0.28	0.000	700
Suzuki 2018	0.04	0.03 , 0.06	0.000	470
Utaile 2023	0.48	0.46 , 0.51	0.117	1535
Velasco (a) 2014	0.08	0.06 , 0.10	0.000	779
Velasco (b) 2014	0.21	0.18 , 0.24	0.000	779
Watson 2013	0.15	0.13 , 0.17	0.000	1726
Zapata-Calvente 2022	0.09	0.07 , 0.12	0.000	592
Zheng 2020	0.16	0.14 , 0.19	0.000	813
Overall (random-effects model)	0.26	0.22 , 0.30	0.000	70860

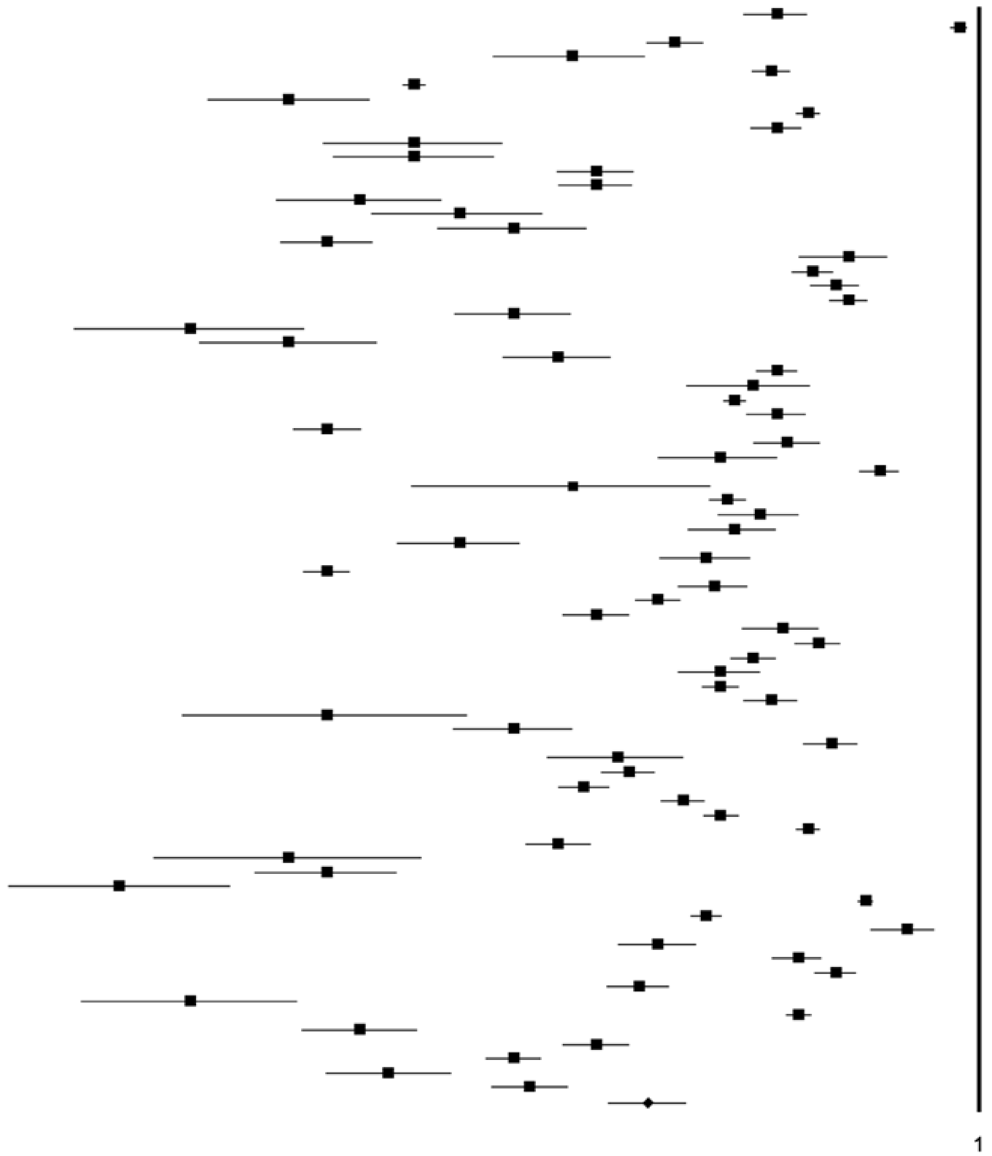

b)

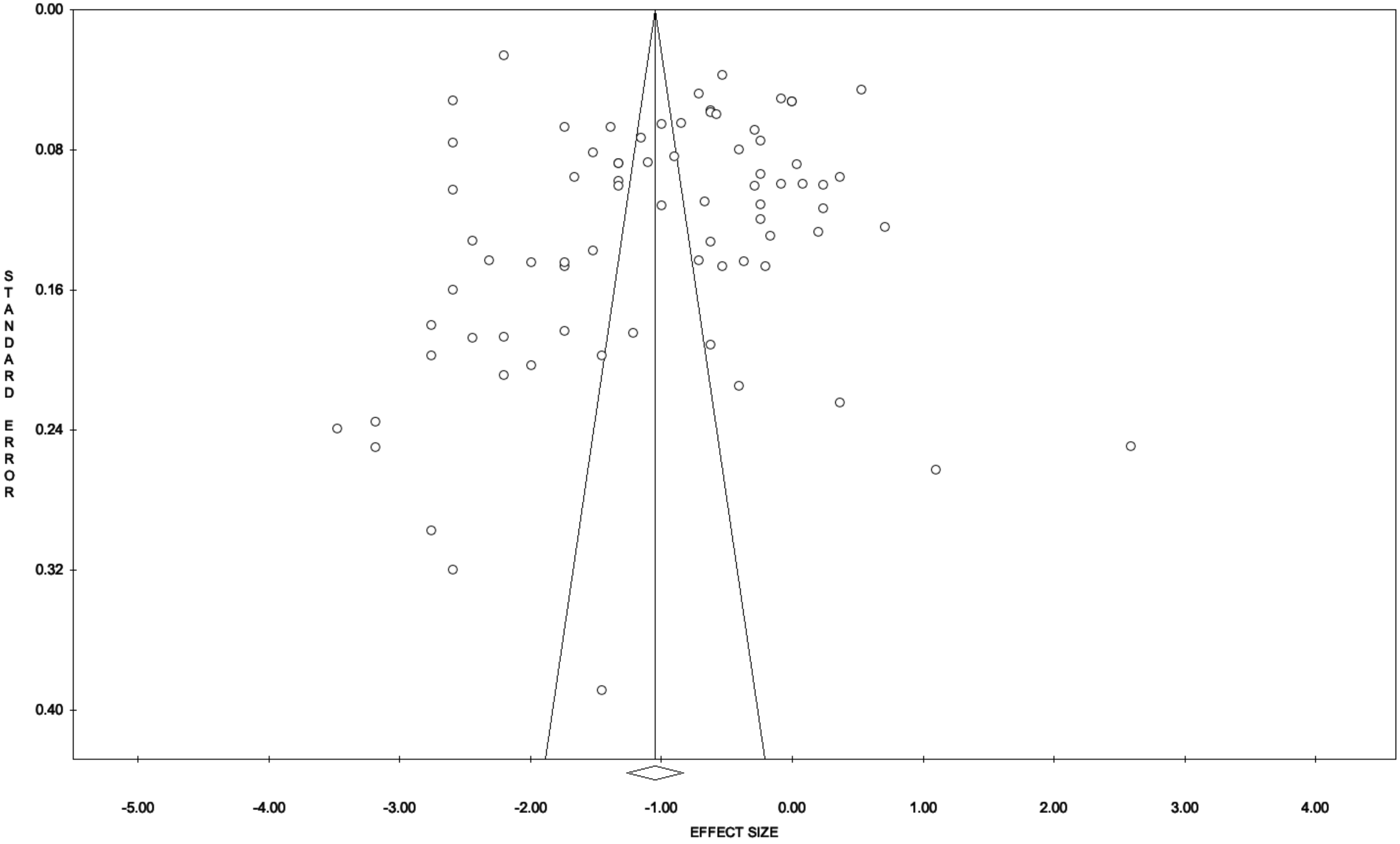

Interpretive note:

The forest plot (Figure S4a) shows prevalence estimates of any intimate partner violence (IPV) across included studies, with moderate variability and a significant pooled effect size (event rate of 0.26, 95% CI: 0.22–0.30, $p < 0.001$). The funnel plot (Figure S4b) reveals moderate asymmetry, suggesting possible small-study effects or publication bias. Together, these figures support the overall robustness of the pooled estimate while indicating some heterogeneity in study precision.

Table S2. Summary statistics of random-effects and fixed-effects models.

Summary statistics							Publication bias	
**Analysis**		**Studies included**	**No. of participants**	**df**	**ES (95% CI); p-value**	**I2; p-value**	**Intercept; p-value**	**Estimated ES; p-value**
Type of IPV	Physical IPV	80	63,429	80	ER(fixed)= 0.15 (0.15–0.16); < 0.001<br>ER(random)= 0.10 (0.08–0.12); < 0.001	98.56; 0.001	-6.83; 0.001	0.15; < 0.001
	Psychological IPV	66	55,361	68	ER(fixed)= 0.30 (0.30–0.31); < 0.001<br>ER(random)= 0.26 (0.22–0.31); < 0.001	99.07; < 0.001	-3.16; 0.256	0.30; < 0.001
	Sexual IPV	63	44,284	62	ER(fixed)= 0.16 (0.15–0.16); < 0.001<br>ER(random)= 0.09 (0.07–0.11); < 0.001	98.15; < 0.001	-6.79; < 0.001	0.16; < 0.001
	Any IPV	71	70,86	75	ER(fixed)= 0.28 (0.27–0.28); < 0.001<br>ER(random)= 0.26 (0.22–0.30); < 0.001	99.21; < 0.001	0.46; 0.860	0.28; <0.001
	Verbal IPV	8	7,878	7	ER(fixed)= 0.36 (0.34–0.38); < 0.001<br>ER(random)= 0.16 (0.05–0.40); < 0.001	99.26; < 0.001	-15.12; 0.130	0.36; <0.001
	Economic IPV	7	2,143	6	ER(fixed)= 0.27 (0.25–0.29); < 0.001<br>ER(random)= 0.13 (0.06–0.27); < 0.001	97.86; < 0.001	-12.27; 0.018	0.27; <0.001

Table S3. Summary statistics of random-effects and fixed-effects models: Sensitivity analyses by WHO region.

Summary statistics							Publication bias	
**Analysis**		**Studies included**	**No. of participants**	**df**	**ES (95% CI); p-value**	**I2; p-value**	**Intercept; p-value**	**Estimated ES; p-value**
WHO Region	African	22	13,190	19	ER(fixed)= 0.42 (0.41–0.43); < 0.001<br>ER(random)= 0.37 (0.30–0.44); < 0.001	98.47; < 0.001	-6.70; 0.139	0.34; < 0.001
	Americas	16	10,0034	10	ER(fixed)= 0.36 (0.35–0.37); < 0.001<br>ER(random)= 0.22 (0.14–0.33); < 0.001	99.10; < 0.001	-10.80; 0.051	0.22; < 0.001
	Eastern Mediterranean	16	3,623	10	ER(fixed)= 0.45 (0.44–0.47); < 0.001<br>ER(random)= 0.51 (0.42–0.59); 0.904	95.96; < 0.001	-8.62; 0.103	0.51; 0.904
	European	15	21,543	10	ER(fixed)= 0.14 (0.13–0.14); < 0.001<br>ER(random)= 0.16 (0.10–0.26); < 0.001	99.13; < 0.001	5.56; 0.298	0.16; <0.001
	South-East Asia	15	4,771	8	ER(fixed)= 0.21 (0.20–0.22); < 0.001<br>ER(random)= 0.18 (0.15–0.23); < 0.001	90.98; < 0.001	-2.92; 0.253	0.18; <0.001
	Western Pacific	13	15,274	11	ER(fixed)= 0.17 (0.16–0.17); < 0.001<br>ER(random)= 0.13 (0.08–0.21); < 0.001	99.16; < 0.001	-3.42; 0.621	0.13; <0.001

Table S4. Summary statistics of random-effects and fixed-effects models: Sensitivity analyses by country income level.

Summary statistics							Publication bias	
Analysis		Studies included	No. of participants	df	ES (95% CI); p-value	I2; p-value	Intercept; p-value	Estimated ES; p-value
Country income level	High	25	34,213	18	ER(fixed)= 0.13 (0.13–0.13); < 0.001	99.03; < 0.001	5.31; 0.187	0.17; < 0.001
					ER(random)= 0.17 (0.12–0.23); < 0.001			
	Upper middle	33	14,874	24	ER(fixed)= 0.36 (0.35–0.36); < 0.001	98.18; < 0.001	-8.23; 0.003	0.26; < 0.001
					ER(random)= 0.26 (0.21–0.31); < 0.001			
	Lower middle	32	17,549	25	ER(fixed)= 0.36 (0.35–0.37); < 0.001	98.65; < 0.001	-4.04; 0.285	0.31; < 0.001
					ER(random)= 0.31 (0.25–0.38); < 0.001			
	Low	8	4,224	5	ER(fixed)= 0.44 (0.42–0.45); < 0.001	97.04; < 0.001	-6.29; 0.516	0.37; 0.009
					ER(random)= 0.42 (0.33–0.51); 0.097			
